# PKR kinase directly regulates tau expression and Alzheimer's disease‐related tau phosphorylation

**DOI:** 10.1111/bpa.12883

**Published:** 2020-08-06

**Authors:** Lasse Reimer, Cristine Betzer, Rikke Hahn Kofoed, Christiane Volbracht, Karina Fog, Chaitanya Kurhade, Emma Nilsson, Anna K. Överby, Poul Henning Jensen

**Affiliations:** ^1^ Danish Research Institute of Translational Neuroscience – DANDRITE Aarhus University Aarhus Denmark; ^2^ Department of Biomedicine Aarhus University Aarhus Denmark; ^3^ H. Lundbeck A/S Valby Denmark; ^4^ Department of Clinical Microbiology, Virology Umeå University Umea Sweden; ^5^ Laboratory for Molecular Infection Medicine Sweden (MIMS) Umeå University Umea Sweden

**Keywords:** neurodegeneration, neuroinflammation, PKR, Tau, tauopathies

## Abstract

Deposition of extensively hyperphosphorylated tau in specific brain cells is a clear pathological hallmark in Alzheimer's disease and a number of other neurodegenerative disorders, collectively termed the tauopathies. Furthermore, hyperphosphorylation of tau prevents it from fulfilling its physiological role as a microtubule‐stabilizing protein and leaves it increasingly vulnerable to self‐assembly, suggestive of a central underlying role of hyperphosphorylation as a contributing factor in the etiology of these diseases. Via *in vitro* phosphorylation and regulation of kinase activity within cells and acute brain tissue, we reveal that the inflammation associated kinase, protein kinase R (PKR), directly phosphorylates numerous abnormal and disease‐modifying residues within tau including Thr181, Ser199/202, Thr231, Ser262, Ser396, Ser404 and Ser409. Similar to disease processes, these PKR‐mediated phosphorylations actively displace tau from microtubules in cells. In addition, PKR overexpression and knockdown, respectively, increase and decrease tau protein and mRNA levels in cells. This regulation occurs independent of noncoding transcriptional elements, suggesting an underlying mechanism involving intra‐exonic regulation of the tau‐encoding microtubule‐associated protein tau (MAPT) gene. Finally, acute encephalopathy in wild type mice, induced by intracranial Langat virus infection, results in robust inflammation and PKR upregulation accompanied by abnormally phosphorylated full‐length‐ and truncated tau. These findings indicate that PKR, independent of other kinases and upon acute brain inflammation, is capable of triggering pathological modulation of tau, which, in turn, might form the initial pathologic seed in several tauopathies such as Alzheimer's disease and Chronic traumatic encephalopathy where inflammation is severe.

## Introduction

With a progressively older population, the prevalence of age‐associated neurodegenerative disease has increased. In a number of these diseases, together named tauopathies, the major neuronal microtubule‐assembly‐promoting protein, tau, plays a significant role (30). Alzheimer's disease (AD) is the most predominant tauopathy and the two main histopathological hallmarks in this disease are the presence of amyloid‐β plaques and neurofibrillary tangles (NFT) in the brain of the diseased, with the latter consisting of aggregated and hyperphosphorylated tau (23, 63). Tau is encoded by a single gene, microtubule‐associated protein tau (MAPT), but exists in six isoforms containing zero (0N) to two (2N) amino‐terminal inserts and three (3R) or four (4R) microtubule‐binding domains (20, 30). Tau isolated from individuals with AD is extensively phosphorylated and more than 40 sites have been reported so far (1, 25, 26). Hyperphosphorylated tau has an impaired ability to stabilize microtubules, and is capable of self‐assembling into aggregates that can template normal tau into filaments, making hyperphosphorylation a clear disease‐determining posttranslational modification in tauopathies (2, 3, 4, 14, 29, 41).

In neurons, a key part of the defense against viral and bacterial infections is undertaken by the 551 amino acid protein Protein kinase R (PKR), also known as eukaryotic translation initiation factor 2‐alpha kinase 2 (EIF2AK2) (11, 44). PKR is an ubiquitously expressed serine‐threonine kinase that is activated either by direct binding to viral dsRNA or indirectly via bacterial lipopolysaccharide or specific pro‐inflammatory cytokines such as Tumor Necrosis Factor alpha (TNF‐α), Interleukin‐1 (IL‐1) and Interferon (IFN)‐γ (5, 19, 27, 40, 56, 60). Furthermore, PKR expression is elevated via an IFN‐stimulated response element within the PKR encoding gene (37). Activation of human PKR, has been linked to different neurologic disorders like Parkinson's disease (PD), Huntington's disease (HD) and AD (8, 10, 42, 43, 46, 49, 51), and inhibition of PKR attenuates neuronal loss, motor deficits and memory deficits in AD mice models (48, 58). Active PKR co‐localizes with abnormally phosphorylated tau in AD brains and was recently found to phosphorylate another protein involved in neurodegenerative diseases, namely α‐synuclein (9, 10, 50, 53), thus raising the question; can PKR also directly phosphorylate tau? Treatment of cortical neurons with the protein phosphatase‐2A inhibitor okadaic acid increased PKR, PKR‐like endoplasmic reticulum kinase (PERK) and general control nonderepressible 2 (GCN2) activation and tau Ser396 phosphorylation (32). In addition, tunicamycin and Aβ treatment induce Glycogen synthase kinase (GSK)‐3β activation in a PKR‐dependent manner in SH‐SY5Y cells, leading to increased tau phosphorylation (9). However, a thorough investigation of PKRs direct role in phosphorylating tau has to date never been done.

In this study, we demonstrate that PKR directly can phosphorylate at least seven tau sites independent of GSK3β, and thereby displace tau from microtubules. In addition, PKR seems to play an indirect role in controlling additional phosphorylation sites. Further, tau mRNA and protein levels are regulated by the presence of PKR. Finally, acute encephalitis, induced by intracranial brain injection of Langat virus in wild‐type (wt) C57BL/6 mice, leads to PKR upregulation and abnormal AD‐associated tau phosphorylation in the brain. Together, these findings suggest that PKR activation upon brain infection or inflammation could participate in the etiology of AD and other tau‐related disorders.

## Results

### PKR directly targets numerous tau phosphorylation sites independent of GSK3β

**Figure 1:**
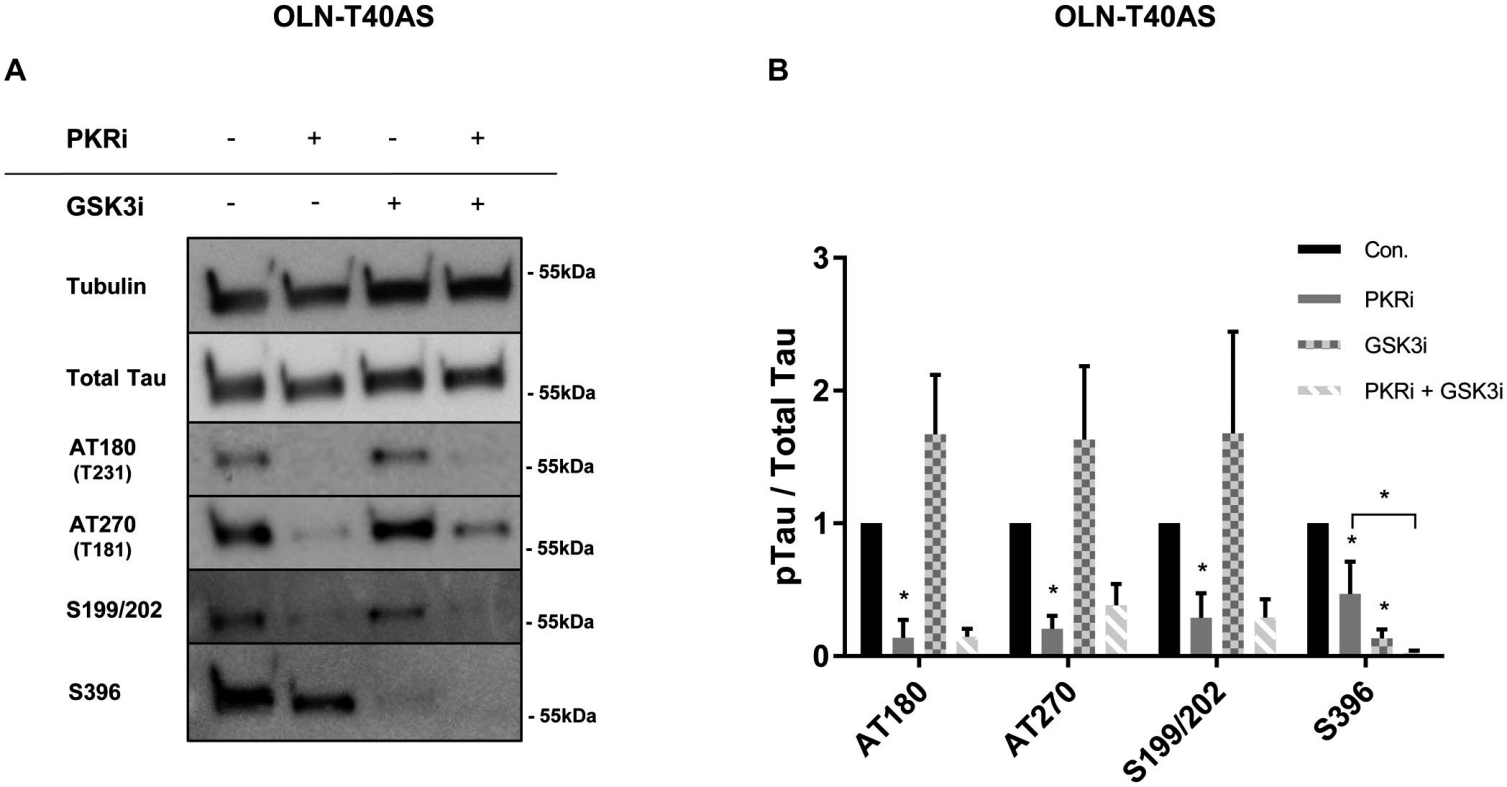
*PKR inhibition decreases tau phosphorylation at several residues independent of GSK3β*. **A.** Immunoblot of whole‐cell lysates from OLN‐T40AS cells using total‐ and pTau‐specific antibodies. Cells were treated with 10 μM PKRi, 10 μM GSK3i or DMSO as vehicle control for 4 h. **B.** Quantifications of the effect of PKR‐ and GSK3 inhibition on different phospho‐tau epitopes/total tau from three independent experiments (n = 3, *P < 0.05, student *t*‐test).

Given the recent finding, that PKR is capable of phosphorylating the PD‐associated Serine 129 α‐synuclein residue and the fact that α‐synuclein and tau both belong to the group of intrinsically disordered proteins and both become extensively phosphorylated in distinctive neurodegenerative disorders (47, 53), we sought to investigate a potential role for a direct influence of PKR on tau phosphorylation. In cells that stably overexpress the longest human isoform of tau, 2N4R tau and present modifiable phosphorylations on Thr231 (AT180), Thr181 (AT270), Ser199/Ser202 and Ser396 we investigated the effect of individual or dual PKR‐, GSK3‐ inhibition (Figure 1A). We observed that PKR inhibition lowered the phosphorylation on all tested phosphorylation sites, whereas GSK3 inhibition only removed the tau phosphorylation on one site (Ser396). Phosphorylation of the three remaining sites (Thr231, Thr181 and Ser199/Ser202) even trended toward an increase, upon GSK3 inhibition. Taken together, this suggests, that PKR can contribute to tau phosphorylation at several residues independent of GSK3β activation (Figure 1B).

**Figure 2:**
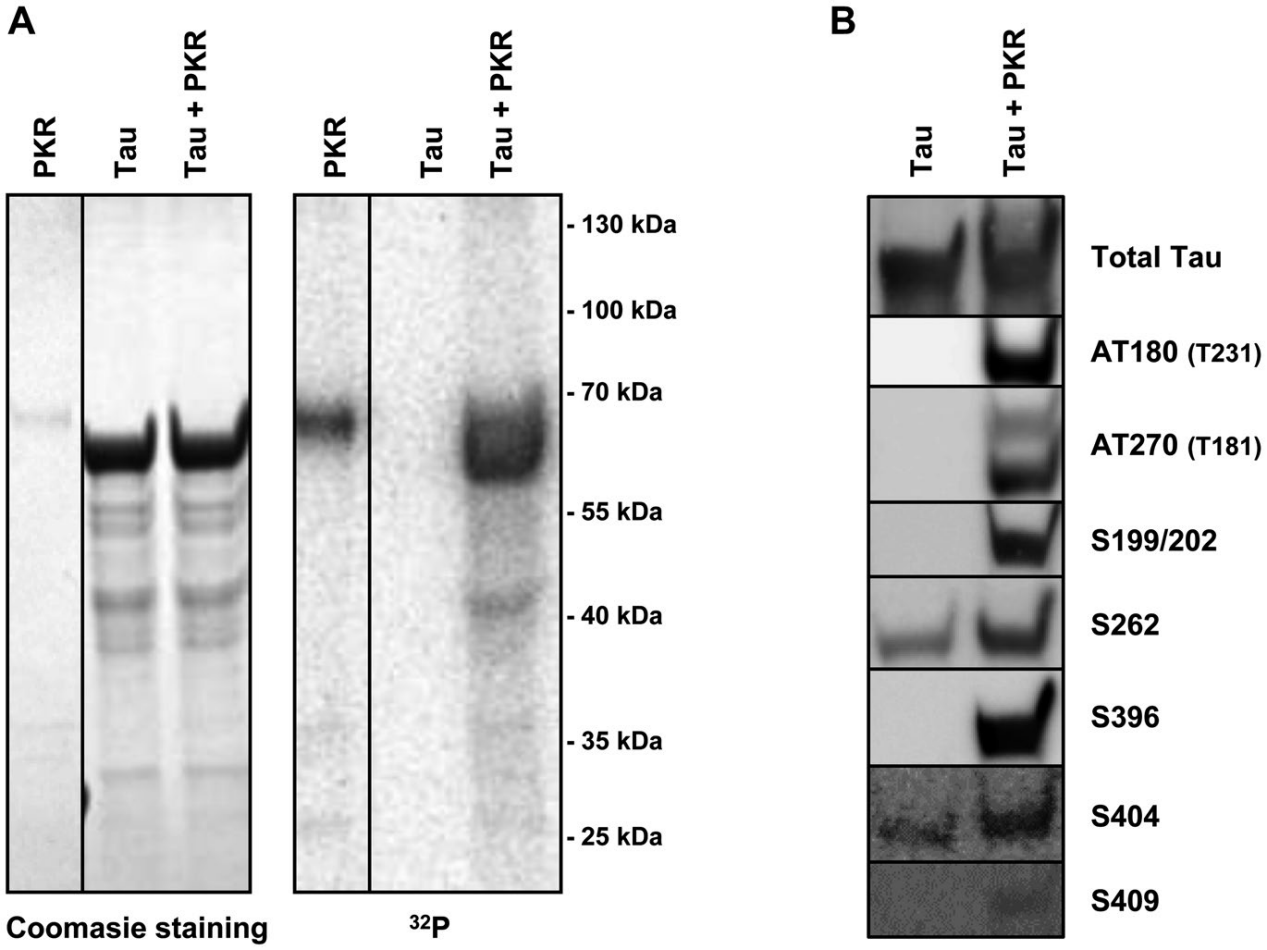
*In vitro phosphorylation demonstrates direct tau phosphorylation by PKR*. **A.** Coomassie Blue stained gel of in vitro phosphorylated samples (left). Recombinant human tau (lane 2 and 3) was incubated for 1 h at 30 °C in phosphorylation buffer, with a 8:1 mixture of non‐labeled and (γ‐32)‐labeled ATP (10 μM final concentration). Tau was incubated alone (lane 2) or with recombinant PKR (lane 3). PKR incubated without tau was included in the experiment (lane 1). ^32^P signal from phosphorylated proteins using standard autoradiography (right). Activation of PKR was evident through autophosphorylation (lane 1 and 3, approximately 70 kDa). PKR also phosphorylated tau (lane 3, approximately 60 kDa). **B.** Immunoblot of recombinant tau using total‐ and pTau‐specific antibodies. Tau was incubated for 18 h at 37 °C in activity phosphorylation buffer with ATP, and without or with recombinant PKR in a 10:1 tau:PKR ratio. 0.25 μg tau was analyzed by immunoblotting. Figure is representative of three independent experiments.

To assess whether PKR directly acts on tau, or if the reduced tau phosphorylation upon PKR inhibition is due to inactivation of specific downstream kinases other than GSK3β, we performed an *in vitro* phosphorylation assay using ^32^P‐labeled ATP and pure recombinant PKR and 2N4R tau proteins (Figure 2A). Coomassie blue staining prior to phospho‐imaging confirmed the purity of both the kinase and substrate (Figure 2A left). Interestingly, PKR phosphorylated tau directly and no detectable labeling occurred when tau was incubated with ^32^P‐ATP alone (Figure 2A right). The activity of PKR was evident through auto‐phosphorylation (Figure 2A right).

To determine if PKR targeted specific AD related epitopes within tau, we performed immunoblotting on *in vitro* phosphorylation tau using different phospho‐specific antibodies. This procedure confirmed five direct PKR‐mediated phosphorylation sites of tau; namely the Thr231, Thr181, Ser199/202, Ser396 and Ser409 epitopes (Figure 2B). An additional two antibodies against the phospho‐Ser262 and ‐Ser404, displayed some background recognition to non‐phosphorylated tau, but the signal was enhanced by incubation with PKR (Figure 2B). PKR had no or little effect on the remaining tested sites Ser202/Thr205 (AT8), Thr212/Ser214 (AT100) and Ser422 (not shown). Of note, some cross‐reactivity with PKR was observed when using the Thr181 tau antibody (Figure 2B).

### PKR inhibition reduces phosphorylation of soluble tau in the brains of transgenic rTg4510 tau mice

**Figure 3:**
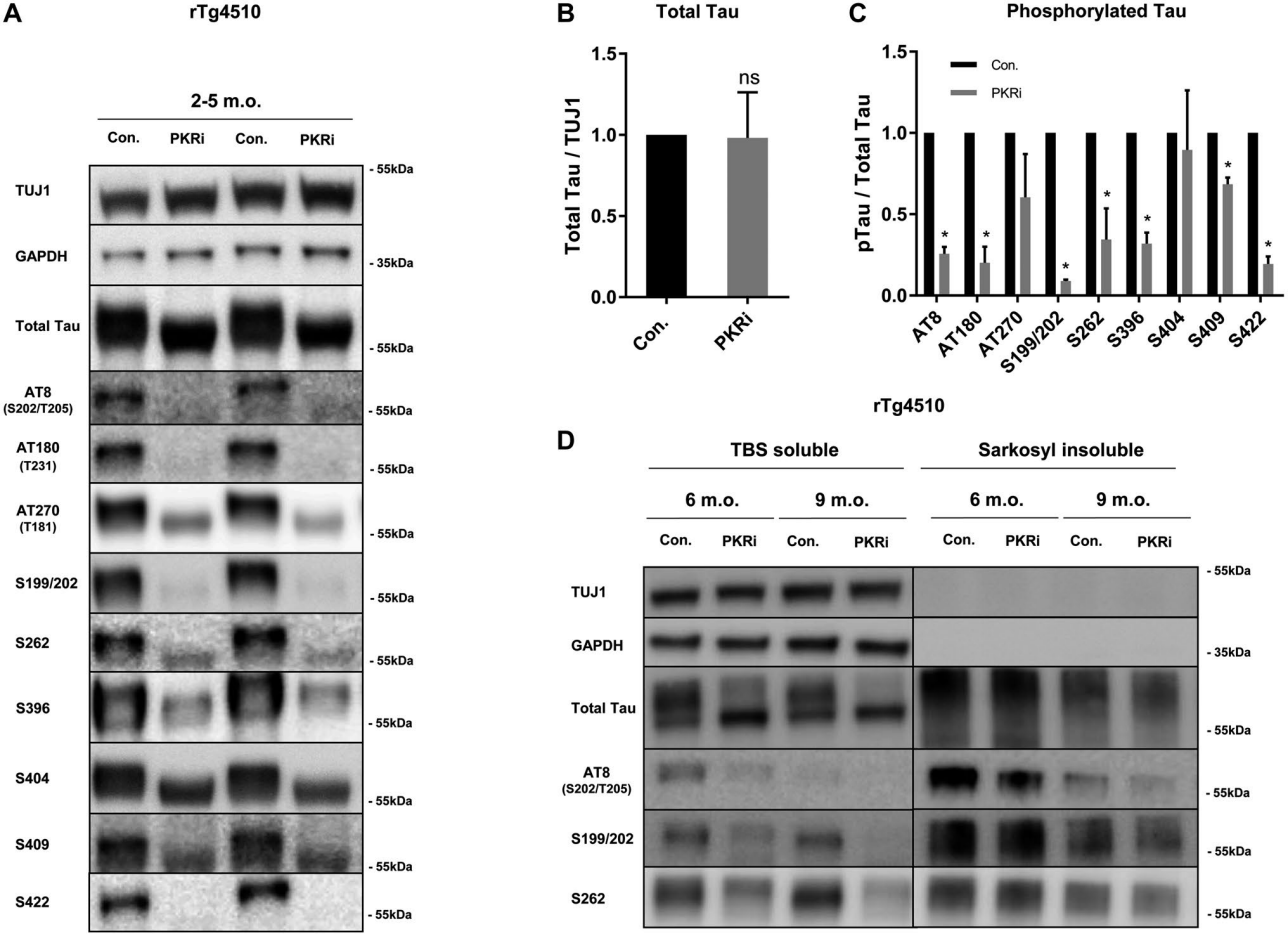
*PKR inhibition decreases soluble tau phosphorylations in transgenic rTg4510 mice brain slices*. **A.** Immunoblot of RIPA‐buffer extracts of acute brain slices using total‐ and pTau‐specific antibodies. Acute brain slices from transgenic rTg4510 mice 2–5 months of age were treated for 4 h with 15 μM PKRi or DMSO as vehicle control. **B and C.** Quantifications of the effect of PKR inhibition on total tau/TUJ1 (B) or phospho‐tau epitopes/total tau (C) from three independent experiments of brain slices prepared from transgenic rTg4510 mice 2–5 months of age. For each experiment two technical replicates were performed (n = 3 biological replicates, *P < 0.05, student *t*‐test). **D.** Immunoblot of acute brain slices from transgenic using total‐ and pTau‐specific antibodies. Acute brain slices from rTg4510 mice of 6‐ (left) or 9 months of age (right) were treated for 4 h with 15 μM PKRi or DMSO as vehicle control. Before immunoblotting brain slices were fractionated into TBS soluble and sarkosyl insoluble fractions.

The well‐described transgenic rTg4510 mice model, overexpressing the human 0N4R P301L mutated tau variant associated with frontotemporal dementia and parkinsonism, displays a large number of disease‐associated tau phosphorylations in the brain already at young age (52, 57, 62). To investigate the role of PKR in these mice, we treated acute brain slice cultures, prepared from 2 to 5 months old rTg4510 mice, with the PKR inhibitor (PKRi) followed by homogenization and immunoblotting (Figure 3). PKR inhibition noticeably condensed the signal of total tau protein into a narrower band, suggesting that several phosphorylations were lost during inhibitor treatment (Figure 3A). However, the overall levels of tau were unaffected by the four hours treatment (Figure 3B).

Indeed, studying individual phosphorylation sites, direct PKR targets Thr231, Ser199/202, Ser262, Ser396 and Ser409 were all reduced by the treatment (Figure 3A,C). Also Ser202/Thr205 and Ser422 phosphorylations, sites that PKR does not directly phosphorylate, were lowered (Figure 3A,C). Although a trend was observed for Thr181, a reduction in phospho‐Thr181 and ‐Ser404 was not significant from these experiments. Treatment with the negative control compound, PKRneg, that mimics PKRi structure, but has no influence on PKR activity, did not reduce the investigated tau phosphorylations (Figure S1A,B).

From 4 months of age and onward, the rTg4510 mice start to accumulate heavily phosphorylated sarkosyl insoluble tau in the brain (52, 62). To assess the effect of PKR inhibition on this pool of phosphorylated tau, we treated acute brain slices, obtained from rTg4510 mice of 6 and 9 months of age, with PKRi and subsequently fractionated the brain homogenate into TBS soluble and sarkosyl insoluble proteins (Figure 3D). PKR inhibition decreased several phosphorylations on TBS soluble tau, but failed to alter the phosphorylations in the sarkosyl insoluble fraction (Figure 3D). Unexpectedly we observed a higher degree of sarkosyl insoluble tau in 6 months old mice compared to 9 months old mice.

### Tau phosphorylation and transcription in cellular systems are regulated by PKR

**Figure 4:**
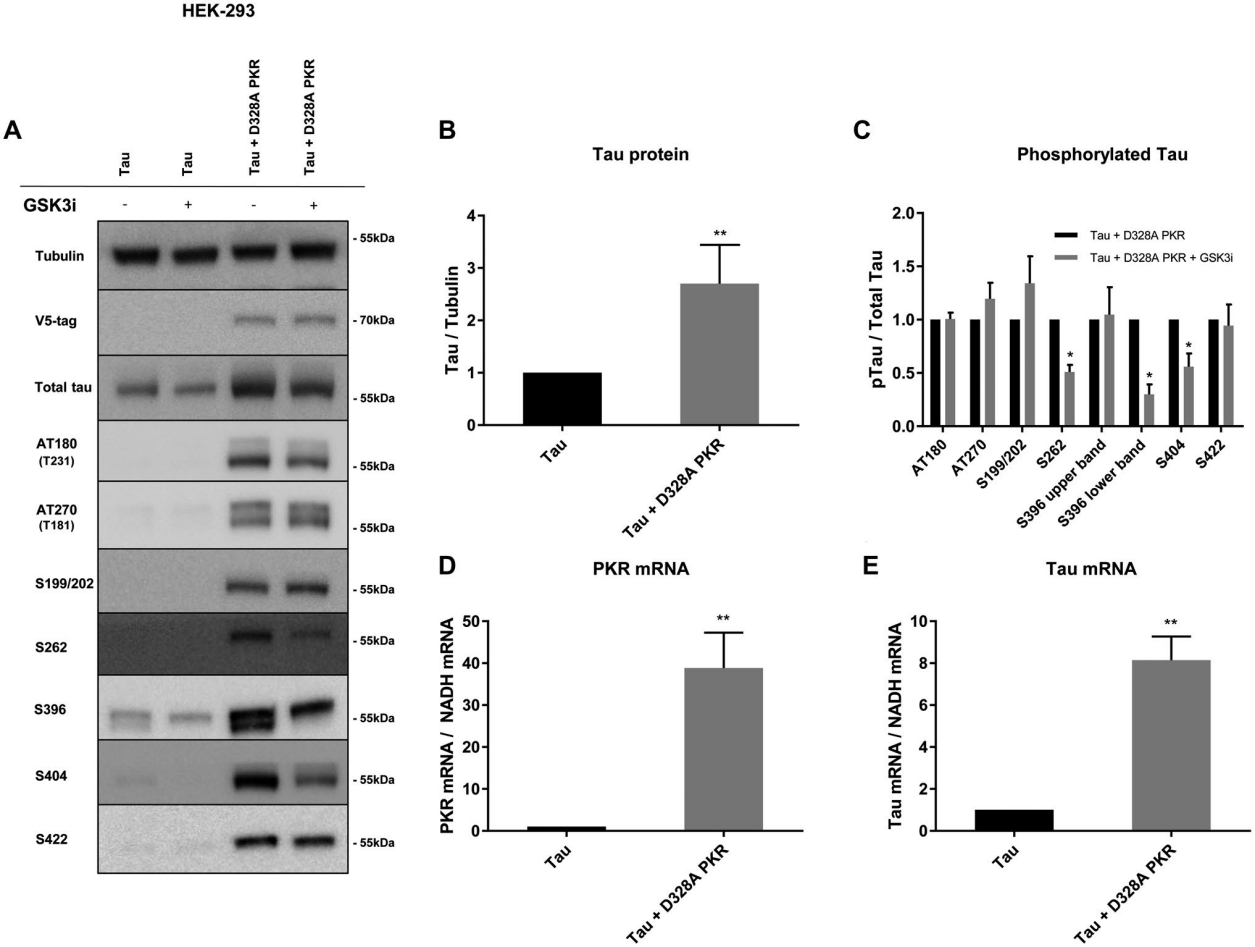
*Overexpression of active PKR increases tau phosphorylation and tau expression*. **A.** Immunoblot of whole‐cell lysates from HEK 293T cells using total‐ and pTau‐specific antibodies as well as anti‐V5 tag to detect V5‐tagged constitutively active D328A PKR. Cells were co‐transfected with vectors expressing 2N4R tau and TD Tomato or 2N4R tau and V5‐tagged constitutively active D328A PKR for 24 h. Cells were treated with GSK3i or DMSO as vehicle control for the last 4 h prior to cell lysis. Figure is representative of five independent experiments. **B.** Quantifications of total tau/α‐tubulin from five independent experiments (n = 5, **P < 0.01, student *t*‐test). **C.** Quantifications of the effect of GSK3 inhibition on different phospho‐tau epitopes/total tau in cells co‐expressing 2N4R tau and V5‐tagged constitutively active D328A PKR. Results from three independent experiments (n = 3, *P < 0.05, student *t*‐test). **D.** mRNA levels in RNA extracts were quantified by qPCR. PKR mRNA levels are normalized to NADH mRNA. The values in the figure exhibit the ratio between cells expressing tau and TD Tomato and cells expressing tau and D328A PKR (n = 4, **P < 0.01, student *t*‐test). **E.** mRNA levels in RNA extracts were quantified by qPCR. Tau mRNA levels are normalized to NADH mRNA. The values in the figure exhibit the ratio between cell expressing tau and TD Tomato and cells expressing tau and D328A PKR (n = 4, **P < 0.01, student *t*‐test).

Small molecule inhibitors have their experimental shortcomings and often exhibit undesirable off‐target effects. Therefore, to further validate the link between PKR activity and tau phosphorylations we chose another strategy: overexpression of 2N4R tau along with TD tomato or the V5‐tagged constitutively active D328A PKR variant (40, 53) in HEK‐293T cells (Figure 4A). In this cell‐line overexpression of the native form of PKR appears inactive as it does not induce phosphorylation of its well‐known target, eif‐2α (40). We therefore opted to use the D328A PKR construct. Interestingly, co‐expression of D328A PKR not only affected tau phosphorylation, but also the level of tau protein. Tau protein levels were almost threefold (2.6 ± 0.8) higher after 24 h in cells co‐expressing D328A PKR, compared to cells co‐expressing control protein TD tomato (Figure 4A,B). For three of the phosphorylation sites the ratio of phosphorylated tau to total tau was increased eightfold (Thr181, 8.0 ± 1.5), twofold upper and lower band (Ser396, 2.1 ± 0.3 and 2.4 ± 0.2) and sixfold (Ser404, 6.2 ± 3.0) by the presence of D328A PKR. Phosphorylation of the remaining tested sites, Thr231, Ser199/202, Ser262 and Ser422 were also elevated by D328A PKR‐expression (Figure 4A), but the fold‐change was not quantifiable since these phosphorylations did not occur in cells where D328A PKR was absent (Figure 4A). These findings suggest that active PKR increase tau phosphorylation on at least seven different phosphorylation sites when overexpressed in cells. No phospho‐tau recognition was observed when overexpressing TD tomato and D328A alone, suggesting that phospho‐tau signals do arise from true tau phosphorylation and not cross‐reactivity with other cellular proteins (Figure S2). Of note, we did observe a faint phospho‐S262‐positive band; however, this was clearly distinguishable from tau (Figure S2).

PKR has previously been suggested to primarily phosphorylate tau via downstream activation of GSK3β. To challenge this general perception, we assessed the effect of GSK3 inhibition in cells expressing tau and D328A PKR (Figure 4C). We observed that only phosphorylations at Ser262, Ser396 lower band and Ser404 were attenuated by GSK3 inhibition, while the remaining sites Thr231, Thr181, Ser199/202, Ser396 upper band and Ser422 were phosphorylated independent of GSKβ activity (Figure 4C). By contrast, PKR inhibition reduced all tau phosphorylations induced by the D328A PKR overexpression (Figure S3A,B). This suggests that D328A PKR is indeed active and directly involved in tau phosphorylation when overexpressed in HEK293T cells. With the exception of the Ser422 epitope, which PKR does not directly target, these findings correlate with *in vitro* phosphorylation data (Figure 2B). Phosphorylation of the Ser202/Thr205 and Ser409 epitopes was not visible under any conditions in this experimental setup (not shown). Together with V5‐positive immunoblotting (Figure 4A), quantitative PCR not only confirmed high overexpression of PKR (Figure 4D), but also revealed an eightfold (8.1 ± 1.1) increase of tau mRNA production in the presence of D328A PKR compared to TD tomato (Figure 4E). This likely explains the observed increase in total tau protein (Figure 4A,B).

**Figure 5:**
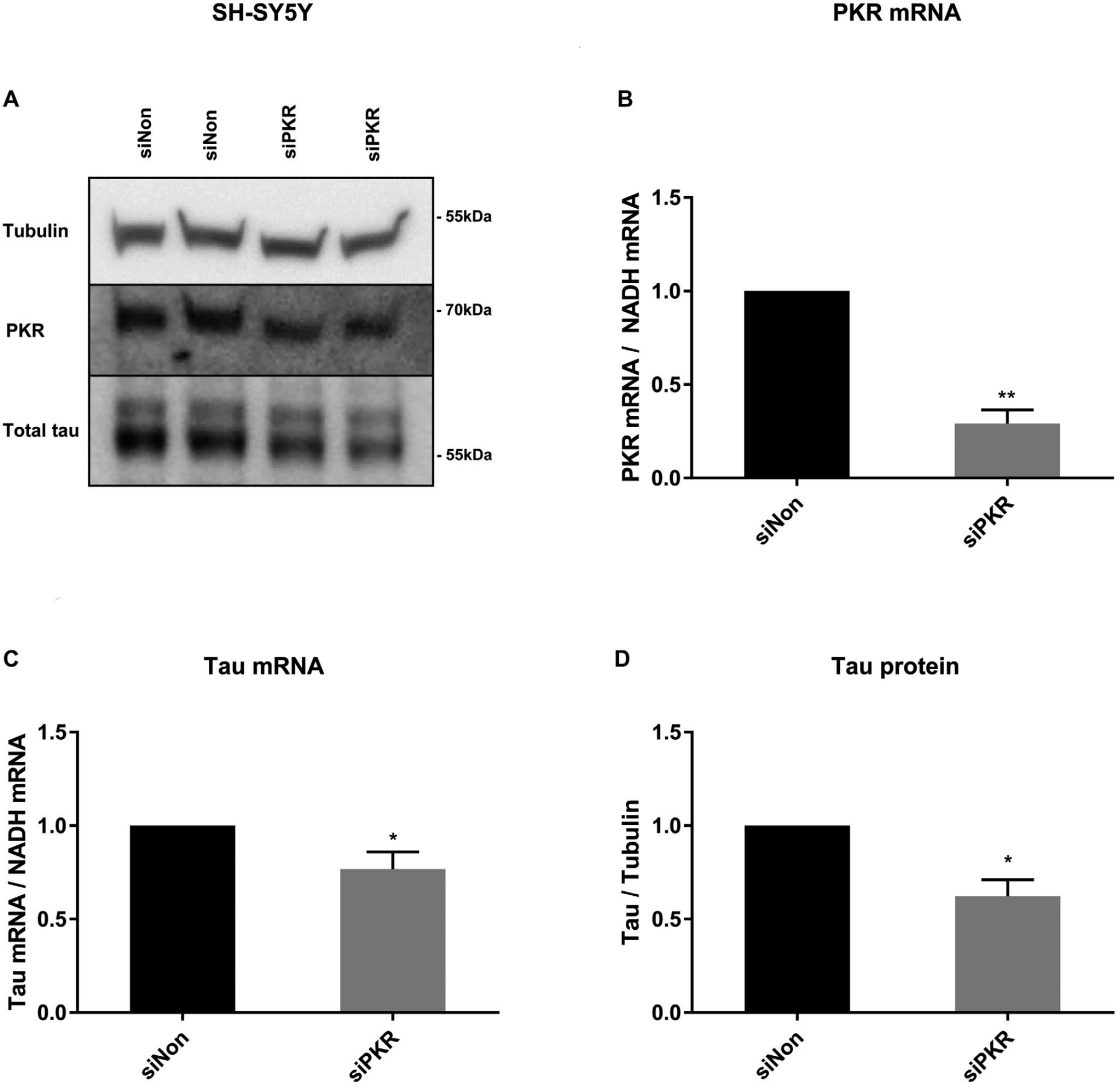
*Silencing of PKR decreases endogenous tau mRNA expression and protein levels in SH‐SY5Y cells*. **A.** Immunoblot of whole‐cell lysate from SH‐SY5Y cells using PKR and total tau antibodies. Cells were transfected with siPKR RNA or non‐interfering siNon RNA for 48 h. prior to analysis. **B.** mRNA levels in RNA extracts were quantified by qPCR. PKR mRNA levels are normalized to NADH mRNA. The values in the figure exhibit the ratio between cell transfected with siNon and siPKR (n = 4, **P < 0.01, student *t*‐test). **C.** mRNA levels in RNA extracts were quantified by qPCR. Tau mRNA levels are normalized to NADH mRNA. The values in the figure exhibit the ratio between cell transfected with siNon and siPKR (n = 4, *P < 0.05, student *t*‐test). **D.** Quantifications of total tau/α‐tubulin from four independent experiments (n = 4, *P < 0.05, student *t*‐test).

Since plasmids used for transient tau expression only contain exons, non‐coding transcriptional regulation of protein transcription might be lost. Therefore, we turned to investigate the PKR‐dependent regulation of endogenous tau expression in SH‐SY5Y cells (Figure 5A). Using siRNA, we successfully lowered endogenous PKR mRNA levels (70.8% ± 7.2%) (Figure 5B). This decrease was followed both by decreased tau mRNA (23.2% ± 9.1%) production (Figure 5C) and lower tau protein levels (37.8% ± 9.0%) in cells where PKR was silenced (Figure 5D), suggesting that PKR regulates transcription of both plasmid based and endogenous tau mRNA expression.

### PKR displace tau from microtubules

**Figure 6:**
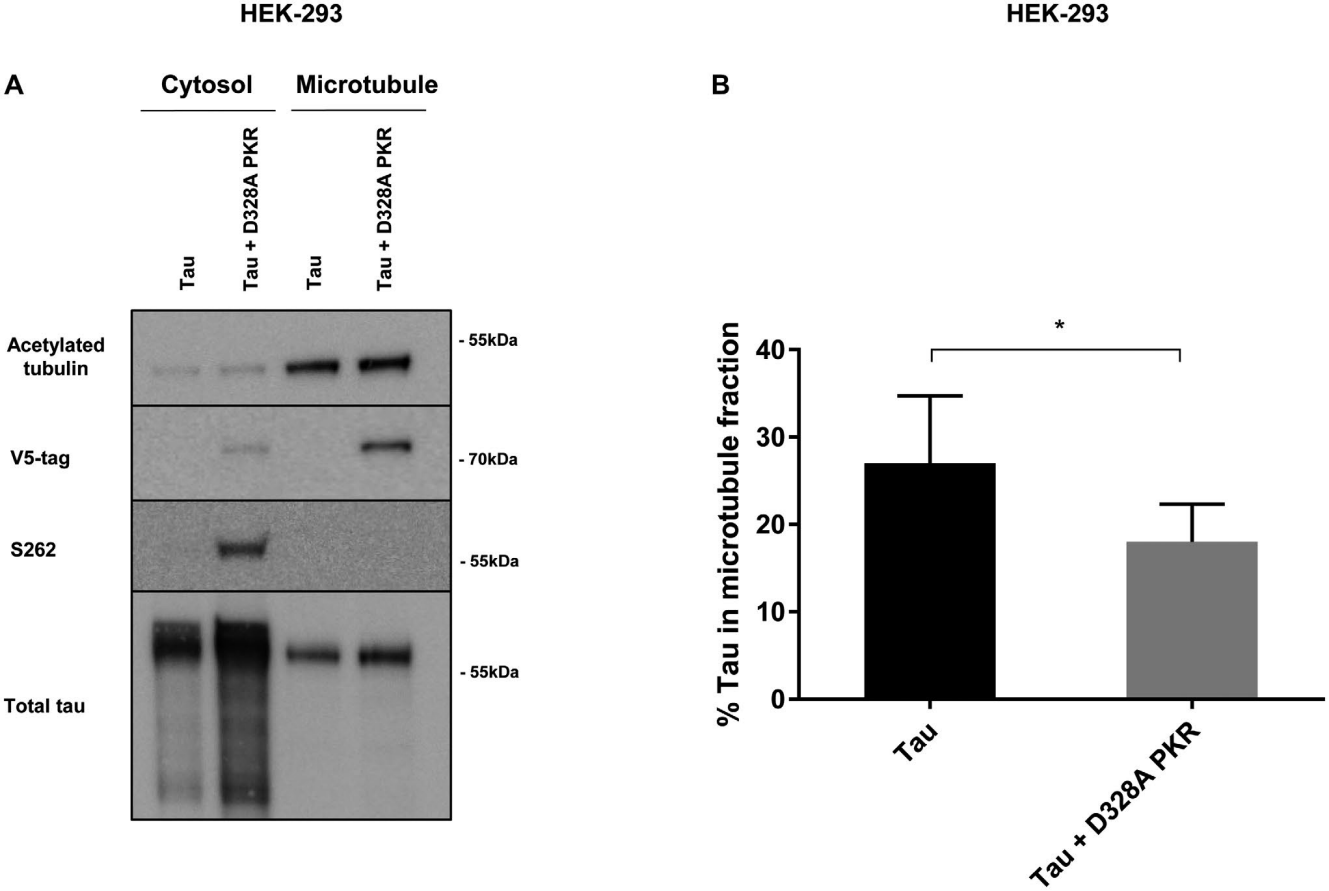
*PKR reduces the affinity of tau to microtubules*. **A.** Immunoblot of HEK293T cells using total‐ and pTau‐specific antibodies as well as anti‐V5 tag to detect V5‐tagged constitutively active D328A PKR. Cells were transfected with 2N4R tau and TD tomato or 2N4R tau and V5‐tagged constitutively active D328A PKR for 24 h. The immunoblot was performed on fractionated samples (cytosol and microtubule fractions). **B.** Quantification of percentage of total tau observed in the microtubule fraction (n = 4, *P < 0.05, student *t*‐test).

Microtubule‐interactions of tau are effectively controlled through phosphorylation, thereby regulating the dynamic instability of microtubules (41, 64). Yet, abnormal and prolonged phosphorylation allow tau to detach from microtubules and leave its subcellular localization in the axon, leading to axonal microtubule instability in neurons (22). Especially Ser262 in the first microtubule binding domain as well as Ser214 and Thr231, significantly reduce microtubule affinity (66). Therefore, we next assessed the consequences of D328A PKR overexpression on tau microbubule‐interactions in HEK293T cells, using a microtubule fractionation assay that separates cytosolic free tau from microtubule‐associated tau (24). We observed that the majority of acetylated tubulin, associated with stable microtubules, was isolated in the microtubule‐fraction as expected (Figure 6A). Simultaneously, phospo‐Ser262 tau was exclusively found within the cytosolic fraction (Figure 6A). These two observations substantiate the robustness of the assay. Quantifying, the relative distribution of tau within each fraction, revealed that the percentage of tau associated with microtubules was significantly lower in the presence of constitutively active PKR (Figure 6A,B). It is worth noting that PKR predominantly is localized in the microtubule fraction (Figure 6A).

### Acute brain infection increases pathology‐associated tau phosphorylations

**Figure 7:**
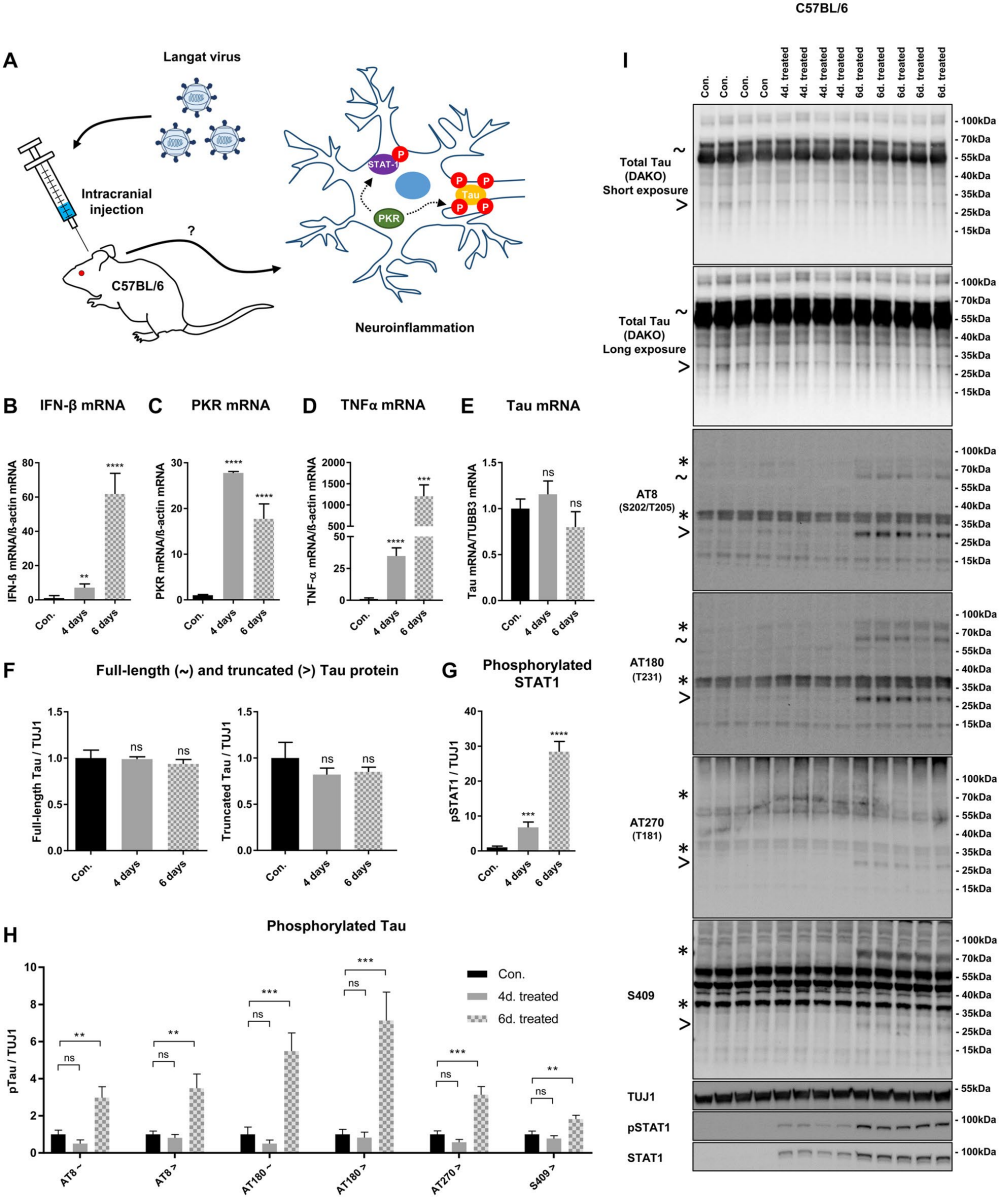
*Brain infection with Langat virus induces PKR upregulation, abnormal tau phosphorylations and tau truncation in C57BL/6J mice*. **A.** Schematic figure describing experimental setup and hypothesis. In brief, C57BL/6J mice were intracranial injected with 100 focus forming units (FFU) of Langat virus for four‐ or six d. PBS injected mice served as control. Langat virus brain infection induces high viral RNA levels and interferon response associated with PKR activation. The hypothesized PKR activation on STAT1 and tau phosphorylation was assessed. **B–E.** mRNA levels in RNA extracted from brain homogenates were quantified by qPCR. IFN‐β mRNA (B), PKR mRNA (C) and TNF‐α mRNA (D) were normalized to β‐actin mRNA while neuronal Tau mRNA (E) was normalized to neuron‐specific class III beta‐tubulin (TUBB3) mRNA. The values in the figure exhibit the ratio between PBS injected mice and 4 or 6 days Langat virus injected mice (n (PBS) = 4, n (4 days Langat virus) = 4 and n (6 days Langat virus) = 5, **P < 0.01, ****<0.0001 student *t*‐test). **F–H.** Quantifications of the effect of 4 or 6 days Langat virus brain infection on total tau/TUJ1 (F), phospho‐STAT1/TUJ1 (G) or full‐length (~) and truncated (>) phospho‐tau epitopes/TUJ1 levels (H) (n (PBS) = 4, n (4 days Langat virus) = 4 and n (6 days Langat virus) = 5, **P < 0.01, ***<0.001, ****<0.0001 student *t*‐test). **I.** Immunoblot of total brain homogenate from C57BL/6J mice intracranial injected with PBS or 100 FFU of Langat virus for 4‐ or 6 days. using total‐ [anti‐tau (A0024, DAKO)] and pTau‐specific antibodies as well as anti‐STAT1 and anti‐pSTAT1. pSTAT1 was used as positive control for PKR activation in the brain. Sign ~ indicates size of total‐ and abnormally phosphorylated full‐length tau, > indicates size of total‐ or abnormally phosphorylated truncated tau species, while * indicates size of non‐tau bands recognized by anti‐mouse secondary antibody.

PKR can be activated directly by viral infections through dsRNA binding or indirectly by specific pro‐inflammatory cytokines and its expression is stimulation by type I interferons (6, 19, 40, 65). Acute encephalitis caused by intracranial Langat virus brain injection in C57BL/6J mice induces high viral RNA levels, substantial inflammation and elevated interferon expression in most brain regions, making it a suitable model to study the effect of PKR activation *in vivo* (Figure 7A) (38). Using this approach, we exposed C57BL/6J wt mice to 4 or 6 days of infection with Langat virus and used PBS‐injected mice as control (Figure 7A). As expected, we observed a high fold increase in IFN‐β mRNA levels four‐ (7.08 ± 2.02) and 6 days (61.76 ± 11.37) post Langat virus injection (Figure 7B). At similar time‐points PKR mRNA (27.76 ± 0.31 and 17.72 ± 3.10 fold, respectively) and PKR‐activating pro‐inflammatory cytokine, TNF‐α mRNA (34.77 ± 6.40 and 1204.95 ± 269.06‐fold, respectively), were also extensively upregulated (Figure 7C,D). Puzzling to our understanding of PKRs role in tau regulation, Langat virus infection did not change tau mRNA brain levels relative to neuronal housekeeping gene neuron‐specific class III beta‐tubulin mRNA (Figure 7E). Neither were any changes observed on total tau protein levels (Figure 7F,I). Yet, relative to ubiquitously expressed β‐actin we detected a decrease in tau mRNA and neuron‐specific class III beta‐tubulin mRNA (Figure S4A,B), both of which neuronal‐expressed proteins, thus, suggesting that neurons start to degenerate as a consequence of Langat Virus brain infection.

Signal transducer and activator of transcription 1 (STAT1) Ser727 phosphorylation is dependent on PKR activity and phosphorylation at this epitope is often used to verify PKR activation (39, 55, 67). We observed that Ser727 STAT1 phosphorylation was elevated in a time‐dependent manner peaking 6 days post injection (Figure 7G,I). Together with the increase in PKR mRNA this suggests elevated levels and activation of PKR upon Langat virus infection. As previously reported, brain infection and specifically, type I IFN signaling also upregulate total STAT1 levels (Figure 7I) (15).

Interestingly, 6 days post Langat virus injection we observed phosphorylation of full‐length mouse tau on residues corresponding to Ser202/Thr205 and Thr231 of human tau (Figure 7H,I). Furthermore, a Ser202/Thr205‐, Thr231‐, Ser409‐ as well as a Thr181 positive ≈28 kDa band was observed (Figure 7H,I). This size does not correspond to any of the six different tau isoforms, but rather suggests a truncated version of tau. Using our regular polyclonal tau antibody raised against 2N4R human tau, we did not detect this species (Figure S5). However, using a different total tau antibody, we successfully recognized the ≈28 kDa tau band (Figure 7I). Proportionally, compared to full‐length tau, the truncated tau species appear more heavily phosphorylated, suggesting that truncated tau poses a better kinase substrate (Figure S6A,B). Of note, two species of roughly 35 and 70 kDa in size, unrelated to phospho‐tau, were detected by secondary mouse antibody alone (Figures 7I and S7). Although PKR did not directly phosphorylate the Ser202/Thr205 epitope in our *in vitro* phosphorylation assay, inhibition of PKR did block Ser202/Thr205 phosphorylation in the transgenic rTg4510 mice brain slices (Figure 3A). This suggests that PKR both directly (Thr231, Thr181 and Ser409) and indirectly (Ser202/Thr205) could partake in the infection‐mediated phosphorylations of tau.

## Discussion

A proposed role for PKR in neurodegenerative disease and in particular tauopathies is not new. Active PKR evidently co‐exists with pathology in AD, PD and HD (8, 50, 51), while PKR inhibition prevent LTP‐ and memory impairment in AD mice models (28).

Although, indirect involvement of PKR in tau phosphorylation has already been suggested (9), we are the first to define a direct relationship between PKR activity and the phosphorylation of several AD‐related tau residues. Bose *et al* observed a strong correlation between tunicamycin and Aβ treatment in SH‐SY5Y cells and subsequent activation of PKR and GSK3β as well as elevated tau phosphorylation on Ser202/Thr205, Thr212/Ser214, Thr231 and Thr181 (9). Further, non‐complete inhibition of PKR, using the peptide inhibitor, PRI, somewhat reverted the effects on Ser202/Thr205, Thr212/Ser214 and Thr181 and simultaneously lowered GSK3β activation (9). Based on these findings, the authors proposed a mechanism wherein PKR regulates tau phosphorylation primarily through downstream activation of GSK3β (9). Utilizing high‐content siRNA screening, another study also revealed a correlation between PKR silencing and reduced Ser262 tau phosphorylation (7).

Our results do not necessarily exclude a pathway where PKR induces tau phosphorylation through downstream activation of GSK3β. Nevertheless, of the phospho‐tau epitopes tested in our experiments, this pathway can only apply for S262, S396, S422 and Ser202/Thr205, and in the case of S262 and S422, GSK3β is not the sole kinase involved (Figures 1, 3, 4 and 7). The remaining sites, S199/S202, Thr231, Thr181, S404 and S409 are all targeted directly by PKR and their phosphorylation occur independent of GSK3β activity (Figures 1, 2 and 4). We also found, that PKR is involved in abnormal tau phosphorylation in brain slices derived from transgenic mice (Figure 3). Yet, PKR inhibition only appears to affect the phosphorylation status of the soluble pool of tau and not sarkosyl‐insoluble tau during the 4 h time‐course of the experiment (Figure 3). This suggests that the sarkosyl‐insoluble pool of tau is either a less accessible substrate for PKR or that it becomes slower dephosphorylated by phosphatases compared to TBS‐soluble tau.

Additionally, PKR‐mediated phosphorylation also plays a role in decreasing microtubule association of tau in cells (Figure 6). Given PKRs specificity for Thr231 and Ser262, both of which important for microtubule‐binding (66), this is not completely unexpected. Still, this information is important, in relation to pathology, since abnormal tau has been shown to leave its subcellular localization in the axon due to loss of microtubule‐binding activity (22).

Finally, we observed that Langat virus brain‐injected C57BL/6J wt mice exhibit PKR upregulation and abnormal tau‐phosphorylation on residues corresponding to human Thr231, S202/S205, Thr181 and Ser409 (Figure 7H,I). In addition to phosphorylation of full‐length tau, we also detected phosphorylation of a truncated tau species (Figure 7H,I). Given the location of the specific phosphorylations N‐terminal truncation appears most plausible. Several N‐terminal cleavage sites have been reported in the human brain and the presence of N‐terminally truncated tau species appear to accumulate with AD disease progression (13). Yet, here we do not observe an increase in the levels of truncated tau (Figure 7F), but rather an increase in abnormal phosphorylation of this species (Figure 7H,I). Future studies, using PKR‐ or GSKβ knockout mice could further validate the underlying pathway leading to Langat virus induced abnormal tau phosphorylations.

Immunolabeling has previously revealed co‐localization of active PKR, active GSK3β and hyper‐phosphorylated tau in postmortem AD brain tissue (9). This could imply a pathway where PKR activates GSK3β, which, in turn, leads to tau hyperphosphorylation as previously proposed. However, GSK3β is activated through Tyr216 phosphorylation and PKR is a serine/threonine kinase, meaning that PKR‐mediated regulation of one or more downstream kinases must precede GSK‐3β activation. We therefore consider it more likely, that tau hyperphosphorylation, upon co‐localization with PKR, is facilitated directly by PKR rather than PKR‐mediated downstream activation of GSK‐3β.

From our observations, PKR evidently also participates in the regulation of tau protein expression (Figures 4 and 5). Since tau mRNA levels align with tau protein levels upon PKR overexpression and silencing, we propose, that this regulation primarily relies on transcription. Remarkably, PKR affects both plasmid‐derived (in HEK‐293T cells) and endogenous (in SH‐SY5Y cells) tau mRNA expression. Our vector used for stable and transient expression in cell‐models only contains the coding sequence, and therefore, no endogenous regulatory elements or promoter regions. This implies that PKR‐mediated mRNA regulation functions independent of non‐coding transcriptional regulators; hence, within the exonic regions. Intra‐exonic transcriptional regulation has previously been described (31) and recently another kinase, polo‐like kinase 2 (PLK2), was found to regulate α‐synuclein mRNA and protein expression via this exact mechanism (34). The observation, that PKR can regulate tau expression, is not entirely new. PKR silencing has previously been associated with decreased 0N4R tau protein levels in stably transfected human H4 neuroglioma cells (7). However, to the best of our knowledge, we are the first to describe PKR regulation of endogenous tau. Further, we propose that this effect is mediated through intra‐exonic transcriptional regulation. Since regulation occurs on both 0N4R tau (7) and 2N4R tau (Figure 4), exonic PKR‐dependent regulatory elements must be located outside exons encoding the amino‐terminal inserts. Yet, more experiments are needed to locate the exact regulatory region within the MAPT gene. We cannot exclude the possibility that PKR also plays a role in stabilization of tau mRNA, however given the rapid regulation observed in our experiments and the high stability of tau mRNA with a reported half‐life of ≈24 h (61), we consider this hypothesis unlikely. In contrast to our cell‐based experiments, no difference in endogenous tau mRNA and tau protein levels was observed upon PKR upregulation in Langat virus infected mice (Figure 7E,F). Yet, a number of reasons could explain this discrepancy. In contrast to our controlled cell studies where we overexpress‐ and silence PKR (Figures 4 and 5), acute virus‐induced encephalitis will initiate a complex series of cell‐ and tissue‐specific events which possibly could counteract the effects of upregulated PKR. Furthermore, our cell experiments rely on endogenous‐ and overexpressed tau of human origin and although human and mouse tau mRNA share almost 86% similarity we do not know whether gaps and 14% disparity between the two species might affect the proposed intra‐exonic transcriptional regulation.

**Figure 8:**
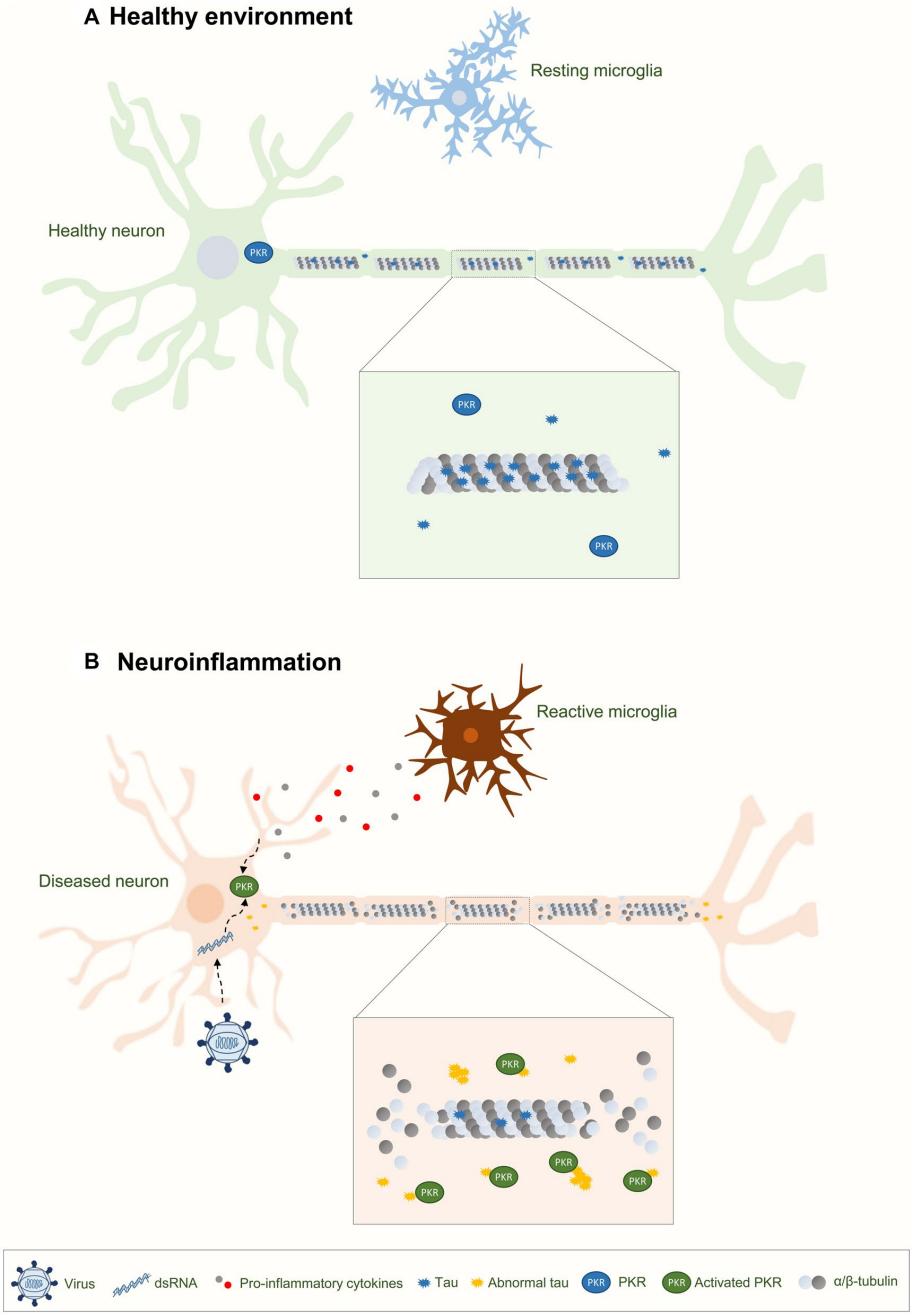
*Involvement of PKR in tau‐dependent neurodegenerative processes*. **A.** Healthy CNS environment. PKR is constitutively expressed at low levels and mainly exists in its inactive state. Tau is associated with the axon where it stabilizes microtubules. **B.** Inflammation due to eg, CTE or systemic and cerebral infections initiates activation of the innate immune response, resulting in glial release of pro‐inflammatory cytokines TNF‐α, IL‐1 and IFN‐γ that activate PKR, and type I interferons involved in enhanced PKR expression. Activation of PKR can directly increase levels of abnormally phosphorylated tau resulting in decreased microtubule binding, increased microtubule instability, re‐localization of tau outside the axon and an increased propensity of tau to undergo self‐assembly into tangles. Such temporally defined periods of inflammation‐induced PKR activation may contribute enough to tip the balance and initiate tau pathology.

Microglia activation has been observed to precede visible tau pathology (70) and a link between pro‐inflammatory cytokine signaling and tau hyperphosphorylation is well‐established (16). PKR activity and expression greatly depend on inflammation. Type I interferons stimulate PKR expression via an IFN‐stimulated response element within the PKR encoding gene (37) and PKR is activated both directly via dsRNA‐binding and indirectly via lipopolysaccharide (LPS), ER stress, specific cytokines such as TNF‐α, IL‐1 and IFN‐γ that are all capable of activating PKR through the PKR activating protein (PACT) that serves as a PKR activating ligand (19, 40, 60). This could explain how herpes simplex virus type 1 (HSV1) infections induce abnormal tau phosphorylation (36, 68) and why chronic periodontitis and porphyromonas gingivalis brain‐levels correlate with AD (17). Furthermore it has been speculated that viral infections and inflammation could leave neurons vulnerable to degeneration, and thereby precede clinical onset of neurodegenerative diseases such as tauopathies (12). If valid, this could have significant impact on the current pandemic outbreak of SARS‐CoV‐2 where initial reports suggest that around one‐third of COVID‐19 patients develop neurological symptoms, including headache, disturbed consciousness and paresthesia (69). SARS‐CoV‐2 has also been detected in cerebrospinal fluid and shown capable of inducing viral encephalitis (69). Whether this will result in a subsequent rise in neurodegenerative disorders and whether PKR plays a significant role in this scenario has yet to be elucidated, but observations from the initial SARS‐CoV suggest that PKR can indeed become activated by coronaviruses (35). Traumatic brain injury (TBI) or chronic traumatic encephalopathy (CTE) where brain inflammation is significant are other diseases where PKR activation might precede tau hyperphosphorylation. In support of this, postmortem brain transcriptome analysis revealed a significant transcriptional upregulation of the PKR‐encoding EIF2AK2 gene in patients suffering from CTE (59). We therefore hypothesize, that PKR activation through pro‐inflammatory insults, for example, TBI or repetitive mild head impacts as well as systemic and cerebral infections initiate abnormal tau phosphorylation. This will dissociate tau from microtubules in a hyperphosphorylated state prone to aggregation and augment the progression of already ongoing pathology as well as initiate new tau‐associated disease in previously healthy neurons (Figure 8). This hypothesis could potentially explain the underlying pathological pathways leading to CTE and dementing in war veterans and retired athletes from physical contact sports such as American football and boxing (18, 21, 45) and the substantial role of inflammation in the progression of AD (33). Based on this hypothesis it will also be interesting to test if PKR phosphorylation facilitates the novel tau filament fold observed in CTE (18).

## Materials and methods

### Cell cultures and treatment

OLN‐93 is an immortalized oligodendroglia cell line derived from primary Wistar rat brain glial cultures (54). OLN‐T40AS cells expressing human neuronal protein α‐synuclein and the longest human tau isoform, 2N4R, were originally made from OLN‐93 oligodendroglial cells and are maintained in 50 µg/mL geneticin at 37°C under 5% CO_2_ and grown in DMEM (Lonza) supplemented with 10% of fetal calf serum, 50 U/mL of penicillin and 50 µg/mL of streptomycin. This cell‐line presents modifiable phosphorylations on Thr181, Ser199/202, Thr231 and Ser396 and is therefore a suitable model for kinase inhibition studies. For experiments, cells were seeded without geneticin 24 h before treatment.

HEK293T cells were cultured as described for OLN‐93, but without geneticin treatment.

For specific experiments, HEK‐ and/or OLN‐T40AS cells were treated with GSK‐3 Inhibitor XV (GSK3i, compound 361558, Calbiochem) or imidazolo‐oxindole PKR inhibitor C16 (PKRi, compound 527450, Merck, Darmstadt Germany) 4 h prior to whole‐cell extraction and immunoblotting.

The SH‐SY5Y cell‐line is a sub‐cloned variant of the human neuroblastoma SK‐N‐SH cell‐line that was kindly provided by Professor, Dr. Leonidas Stefanis, Division of Basic Neurosciences, Biomedical Research Foundation of the Academy of Athens, Athens, Greece. SH‐SY5Y cells were cultured at 37°C under 5% CO_2_ in RPMI 1640 medium with L‐Glutamine (Lonza) supplemented with 15% FCS, 50 U/mL penicillin and 50 μg/mL streptomycin.

### Plasmids, siRNA and transfection

pcDNA3.1 zeo(‐) plasmid expressing human 2N4R tau, TD tomato or human V5‐tagged PKR construct pcDNA3‐PKR D328A (40). The last was a kind gift from Dr. Ganes C. Sen (Cleveland Clinic, Lerner Research Institute). All constructs were confirmed by sequencing. Transient transfections were performed with Lipofectamine 3000 (Thermo Fisher Scientific) transfection reagent according to the manufacturer's protocol.

Small interfering RNA (siRNA) targeting PKR was from Dharmafect (Lafayette, CO, USA). All siRNAs were OnTarget Smartpools containing a mixture of four predesigned sequences used in combination with Lipofectamine 3000 (Thermo Fisher Scientific) transfection reagent as instructed by the manufacturer. Nontargeting siRNA, siNon, was included as control. Cells were used 48 h post siRNA transfection.

### Preparation of whole‐cell extract and immunoblotting

Whole‐cell extracts were prepared by lysing cell pellets in RIPA lysis buffer [50 mM Tris, pH 7.4, 150 mM NaCl, 1% Triton X‐100, 0.5% sodium deoxycholate, 0.1% sodium dodecyl sulfate, 1 mM EDTA, 1 × complete protease inhibitor cocktail (Roche)] with phosphatase inhibitors (25 mM β‐glycerolphosphate, 1 mM Na_3_VO_4_, 10 mM Na‐pyrophosphate, 1 mM NaF) on ice for 30 min and lysates were centrifuged at 25 000 × g for 30 minutes at 4°C. The resulting supernatant was saved as the whole‐cell extract. Protein concentration was determined by bicinchoninic acid assay (Sigma, MO, USA). Whole‐cell extracts were dissolved in loading buffer (4% SDS, 40% glycerol, 1% bromophenol blue and 50 mM Tris, pH 6.8) supplemented with 50 mM dithioerythritol (DTE), denatured at 95°C for 5 minutes, resolved on 10%–16% polyacrylamide gels and proteins transferred onto polyvinylidine difluoride (PVDF) membranes (GE Healthcare, UK). PVDF membranes were blocked in 5% non‐fat milk dissolved in TBST (10 mM Tris, pH 7.4, 150 mM NaCl, 0.1% Tween‐20) with phosphatase inhibitors for 1 h at room temperature (RT), incubated with primary antibodies overnight at 4°C in identical blocking buffer (STAT1 antibodies, were added in 5% BSA TBST with phosphatase inhibitors), washed and incubated with horseradish peroxidase (HRP)‐conjugated secondary antibodies (Dako, Denmark) for 1 h at RT. Proteins were visualized with enhanced chemiluminescence using a Fuji LAS‐3000 Intelligent Dark Box (Fujifilm, Japan). Densitometry was carried out in Image J software (NIH, USA).

### *In vitro* phosphorylation assay

Recombinant human 2N4R tau was resuspended in 2× kinase activity buffer (100 mM Hepes, pH 7.5 20 mM MgCl_2_, 2 mM EGTA) and incubated for 1 h at 37°C with ATP (8:1 mixture of non‐labeled and (γ‐32)‐labeled ATP, 10 µM final concentration) alone or in a 50:1 ratio with recombinant human 62.2 kDa EIF2AK2 (PKR) protein (Thermo Fisher Scientific). The samples were subjected to SDS‐PAGE and stained at RT, using Coomassie Brilliant Blue, destained ON at RT and left for 1 h at RT in a 35% EtOH, 3.5% glycerol solution prior to drying. The dried gel was photographed on Fuji LAS‐3000 Intelligent Dark Box (Fujifilm, Japan) and developed using standard autoradiography.

For phosphorylation of recombinant human 2N4R tau to be subsequently analyzed by immunoblot analysis, *in vitro* phosphorylation was performed using pure, non‐labeled ATP and incubated 18 h at 37°C in 1× kinase activity buffer and subjected to immunoblotting as previously described for whole‐cell extracts.

### Acute brain slices

Acute brain slice preparation was performed as previously described (53). In brief, transgenic rTg4510 mice, overexpressing the 0N4R P301L mutated tau variant (52), were anaesthetized and subsequently decapitated. A 400 μm thick coronal brain slices were prepared in ice‐cold artificial cerebrospinal fluid (ACSF) and kept in ACSF for at least 1 h at RT prior to treatment. Brain slices were divided into hemispheres. One hemisphere was treated with 15 μM PKR inhibitor C16 (PKRi); the other with DMSO (as vehicle control) for comparison for 4 h. During treatment, brain slices were maintained in ACSF at 37°C under 5% CO_2_. Brain slices were dounce‐homogenized in RIPA lysis buffer and left on ice for 30 minutes. Afterward, the lysates were centrifuged at 25 000 × g for 30 minutes at 4°C. The resulting supernatant was saved as brain extract and immunoblotting was performed as described.

For TBS‐ and sarkosyl insoluble tau fractionation, brain slices were treated as above and subjected to fractionation as previously described by Sahara *et al* with minor modifications (57). Hemispheres were homogenized in 10 × volume of TBS homogenization buffer plus protease‐phosphatase inhibitors and PMSF. The homogenate was centrifuged 25 000 × g for 20 minutes, 4°C and divided into supernatant (TBS soluble fraction) and pellet. The pellet was homogenized in buffer B (10 mM Tris, pH 7.4, 0.8 M NaCl, 10% Sucrose, 1 mM EGTA and 1 mM PMSF) and centrifuged 25 000 × g for 20 minutes, 4°C. One percent of sarkosyl (final concentration, stock 10%) was added to the supernatant and incubated at 37°C for 1 h followed by ultracentrifugation, 150 000 × g for 1 h, 4°C. The pellet was resuspended in TE buffer (Sarkosyl Insoluble Fraction). Immunoblotting was performed as described.

### Quantitative PCR determinations

Total RNA purification was performed using High Pure RNA Isolation Kit (Roche Life Science) with an on‐column enzymatic DNA degradation, according to the manufacturer's instructions. RNA concentration was determined on a Nanodrop spectrophotometer and cDNA created using equal amounts of RNA and the TaqMan^®^ Reverse Transcription Reagents with random primers according to manufacturer's instructions (Applied Biosystems). Quantitative PCR was performed using the iTaq^™^ SYBR^®^ Green Mastermix with ROX (BioRad) with the following probes: NADH (NDUFC2, Hs01072843_m1), tau (MAPT, Hs00902194_m1), PKR (EIF2AK2, Hs00169345_m1), β‐actin (ActB, Mm00607939_s1), IFN‐β (Ifnb1, Mm00439552_s1), TNF‐α (Tnf, Mm00443260_g1), tau (MAPT, Mm00521988_m1), neuron‐specific class III beta‐tubulin (Tubb3, Mm00727586_s1) and PKR (EIF2AK2, Mm01235643_m1, all Thermo Fisher Scientific Inc.).

### Cell fractionation into cytosolic and microtubule fractions

Cell fractionation was performed as originally described with small modifications (24). Briefly, an equivalent number of cells co‐expressing tau and TD‐Tomato or tau and PKR D328A were recovered 24 h post transfection in warmed lysis buffer (80 mm Pipes, pH 6.8, 1 mm MgCl2, 2 mm EGTA, 30% glycerol, 0.1% Triton X‐100) supplemented with protease‐ and phosphatase inhibitors. Lysates were ultracentrifuged at 100 000 × g, 27°C for 20 minutes and supernatants collected as cytosolic fraction. The remaining pellet (microtubule fractions) for each sample was washed once and resuspended by sonication in RIPA buffer (supplemented with proteases and phosphatases inhibitors) in an equal volume to that of cytosolic fraction. Samples were mixed with loading buffer and equal volumes analyzed by immunoblotting to assess the relative percentage of tau associated to microtubules within the cells.

### Viral infection of mice

C57BL/6 WT mice were purchased from Harlan laboratories/ENVIGO. *In vivo* infection experiments were performed at Umeå Center for Comparative Biology (UCCB) using Langat virus strain TP21 (a kind gift from Gerhard Dobler, the Bundeswehr Institute of Microbiology, Munich, Germany). For intracranial infections, mice were treated as previously described by Kurhade *et al* (38) In brief, C57BL/6J mice were anesthetized by intraperitoneal injection with a mixture of ketamine (100 μg/g body weight) and xylazine (5 μg/g body weight). Mice were injected in the sagittal suture in between the ears and eyes with 100 focus forming units (FFU) of Langat virus in 20 μL PBS, or PBS alone and sacrificed at indicated time‐points. The injection‐needle is equipped with a stopper to make sure that the needle penetrates equally deep into the brain of all animals. Brains were removed after cardiac perfusion with PBS including phosphatase inhibitors (25 mM β‐glycerolphosphate, 1 mM Na_3_VO_4_, 10 mM Na‐pyrophosphate and 1 mM NaF). Half a brain was dounce‐homogenized in 1 mL PBS including protease‐, phosphatase‐ and RiboLock (Thermo Fisher Scientific) RNAse inhibitor 20 bumps with rotor set at 700 rpm. The homogenates were sonicated (4 × 10 strokes using a Branson Ultrasonics^™^ Sonifier S‐250 A, duty cycle 80 and output control 6), diluted five times and subjected to SDS‐PAGE and immunoblotting.

### Antibodies

Anti‐α‐tubulin (T9026 Sigma‐Aldrich Co.), anti‐neuron‐specific class III β‐tubulin (Tuj1, TUBB3 #80120, BioLegend) anti‐acetylated tubulin (T7451 Sigma‐Aldrich), anti‐GAPDH (MAB374 Merck Millipore) anti‐PKR (anti‐EIF2AK2 Antibody No. ABIN500507, antibodies‐online.com), anti‐V5 tag antibody (SV5‐Pk1, Abcam), anti‐tau antibody (in house produced polyclonal Tau antibody, affinity‐purified rabbit towards human 2N4R tau), *anti‐tau (A0024, DAKO),* anti‐STAT1 (#9172 Cell Signaling Technology), anti‐pSTAT1 (pS727, #9177 Cell Signaling Technology), anti‐pS199/202 tau (44‐768G Thermo Fisher Scientific), anti‐pS396 tau (44‐752G Thermo Fisher Scientific), AT8 (pS202/T205 tau, MN1020 Thermo Fisher Scientific), AT180 (pT231 tau, MN1040 Thermo Fisher Scientific), AT270 (pT181 tau, MN1050 Thermo Fisher Scientific), anti‐pS262 (44‐750G Thermo Fisher Scientific), anti‐pS404 (44‐758G Thermo Fisher Scientific), anti‐pS409 (Lu0041G, kindly provided by Lundbeck) and anti‐pS422 (ab79415, Abcam) were used in accordance with the manufacturers' recommendations. HRP‐conjugated rabbit immunoglobulins (#P0217, Dako) and secondary HRP conjugated mouse immunoglobulins (#P0260, Dako) were used as secondary antibodies.

### Statistics

All comparisons were performed as differences between two or more groups and were analyzed by Student's *t* test for paired data. A *P* value < 0.05 was considered significant.

## Ethical Approval

The mice were maintained under specific pathogen‐free conditions and experiments were approved and carried out according to the guidelines set out by the Regional Animal Ethical Committee (Umeå, Sweden) under approval number A77‐14.

## Conflict of Interests

The authors declare that they have no conflict of interest.

## Supporting information


**Figure S1.** *Negative control compound, PKRneg does not decrease tau phosphorylations in transgenic rTg4510 mice brain slices*. **A.** Immunoblot of RIPA‐buffer extracts of acute brain slices using total‐ and pTau‐specific antibodies. Acute brain slices from transgenic rTg4510 mice 2–5 months of age were treated for 4 h with 15 μM PKRneg or DMSO as vehicle control. **B.** Quantifications of the effect of PKRneg treatment on different phospho‐tau epitopes/total tau from three independent experiments of brain slices prepared from transgenic rTg4510 mice 2–5 months of age. (n = 3, ns = not significant, student *t*‐test).

**Figure S2**. *Correctly sized phospho‐tau detection is only observed in cells overexpressing tau*. Immunoblot of whole‐cell lysate from HEK 293T cells using total‐ and pTau‐specific antibodies as well as anti‐V5 tag to detect V5‐tagged constitutively active D328A PKR. Cells were co‐transfected with vectors expressing TD Tomato and V5‐tagged constitutively active D328A PKR or 2N4R tau and V5‐tagged constitutively active D328A PKR for 24 h. Figure is representative of three independent experiments.

**Figure S3**. *PKR inhibition reverts tau phosphorylations induced by D328A PKR*. **A.** Immunoblot of whole‐cell lysates from HEK 293T cells using total‐ and pTau‐specific antibodies as well as anti‐V5 tag to detect V5‐tagged constitutively active D328A PKR. Cells were co‐transfected with vectors expressing 2N4R tau and TD Tomato or 2N4R tau and V5‐tagged constitutively active D328A PKR for 24 h. Cells were treated with PKRi or DMSO as vehicle control for the last 4 h prior to cell lysis. Figure is representative of three independent experiments. **B.** Quantifications of the effect of PKR inhibition on different phospho‐tau epitopes/total tau in cells co‐expressing 2N4R tau and V5‐tagged constitutively active D328A PKR. Results from three independent experiments (n = 3, *P < 0.05, student *t*‐test).

**Figure S4**. *Brain infection with Langat virus in C57BL/6J mice decreases mRNA from neuronal expressed proteins*. (**A,B**) mRNA levels, in RNA extracted from brain homogenates, were quantified by qPCR. Tau mRNA (A) and neuron‐specific class III beta‐tubulin (TUBB3) mRNA (B) were normalized to β‐actin mRNA. The values in the figure exhibit the ratio between PBS injected mice and four‐ or 6 days Langat virus injected mice (n (PBS) = 4, n (4 days Langat virus) = 4 and n (6 days Langat virus) = 4, **P < 0.01, ****<0.0001 student *t*‐test).

**Figure S5.** *Total tau antibody does not detect truncated species*. Immunoblot of total brain homogenate from C57BL/6J mice intracranial injected with PBS or 100 FFU of Langat virus for 4‐ or 6 days. using in house‐produced total tau antibody.

**Figure S6.** *Truncated tau is more extensively phosphorylated relative to full‐length tau*. Quantifications of full‐length (~) and truncated (>) phospho‐tau epitopes (**A**) AT8 and (**B**) AT180 relative to their respective total tau signal and normalized to full‐length control. Difference in the fraction of phosphorylated full‐length and truncated tau was quantified for PBS‐treated controls, 4 d‐treated and 6 d‐treated mice (n (PBS) = 4, n (4 days Langat virus) = 4 and n (6 days Langat virus) = 5, **P < 0.01, ***<0.001, ****<0.0001 student *t*‐test).

**Figure S7**. *Incubation with secondary mouse antibody alone detects two non‐tau related bands in mice brain homogenates*. Immunoblot of total brain homogenate from C57BL/6J mice using secondary mouse antibody alone. Mice were intracranial injected with PBS or 100 FFU of Langat virus for 4‐ or 6 days. Two species of roughly 35 and 70 kDa in size, unrelated to phospho‐tau, were detected. * indicates size of non‐tau bands recognized by anti‐mouse secondary antibody.

## Data Availability

The data that support the findings of this study are available from the corresponding author upon reasonable request.
