# Supplementary figures and images for: PKR kinase directly regulates tau expression and Alzheimer's disease‐related tau phosphorylation
Source: Brain Pathol. 2020 Aug 6;31(1):103–19. doi: 10.1111/bpa.12883 (PMC8018097; doi:10.1111/bpa.12883)

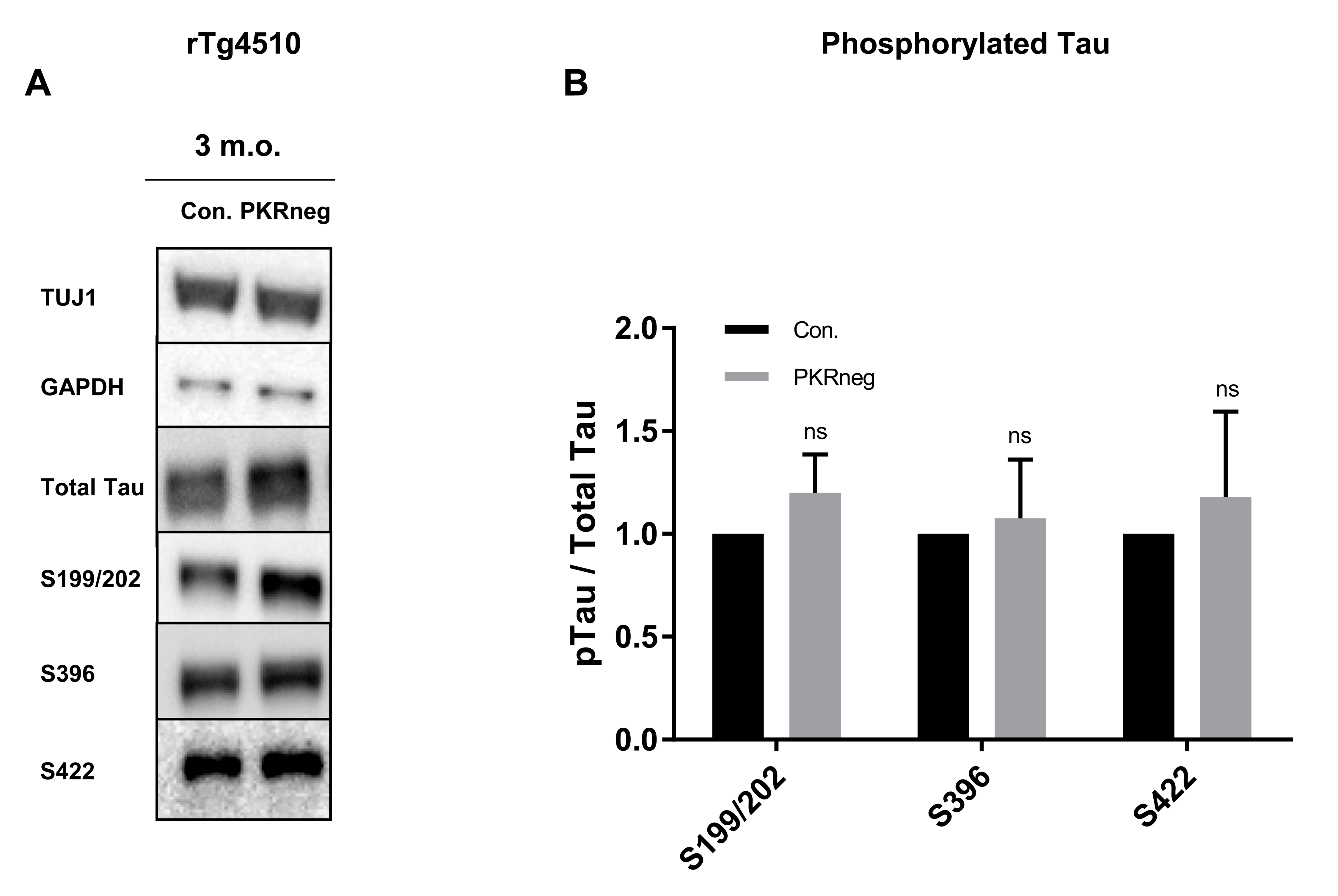

Supplement: Supplementary file 1 — Figure S1. Negative control compound, PKRneg does not decrease tau phosphorylations in transgenic rTg4510 mice brain slices. A. Immunoblot of RIPA‐buffer extracts of acute brain slices using total‐ and pTau‐specific antibodies. Acute brain slices from transgenic rTg4510 mice 2–5 months of age were treated for 4 h with 15 μM PKRneg or DMSO as vehicle control. B. Quantifications of the effect of PKRneg treatment on different phospho‐tau epitopes/total tau from three independent experiments of brain slices prepared from transgenic rTg4510 mice 2–5 months of age. (n = 3, ns = not significant, student t‐test). [file BPA-31-103-s007.tif]

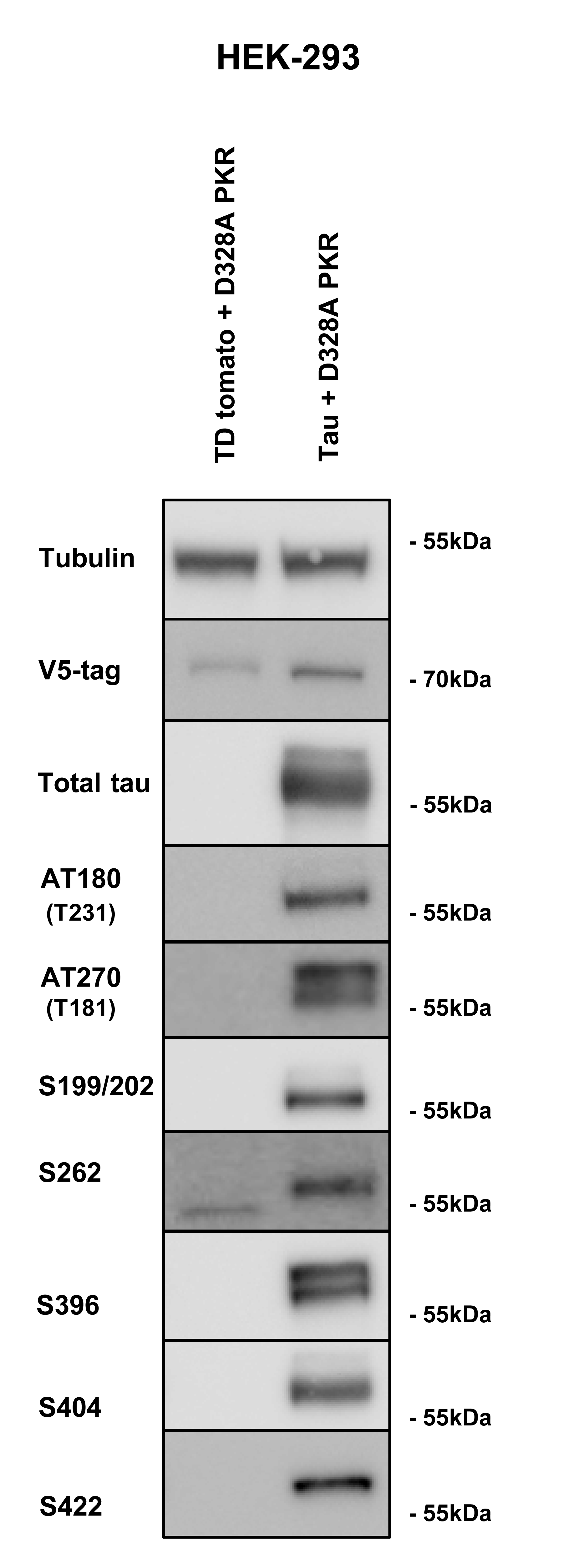

Supplement: Supplementary file 2 — Figure S2. Correctly sized phospho‐tau detection is only observed in cells overexpressing tau. Immunoblot of whole‐cell lysate from HEK 293T cells using total‐ and pTau‐specific antibodies as well as anti‐V5 tag to detect V5‐tagged constitutively active D328A PKR. Cells were co‐transfected with vectors expressing TD Tomato and V5‐tagged constitutively active D328A PKR or 2N4R tau and V5‐tagged constitutively active D328A PKR for 24 h. Figure is representative of three independent experiments. [file BPA-31-103-s006.tif]

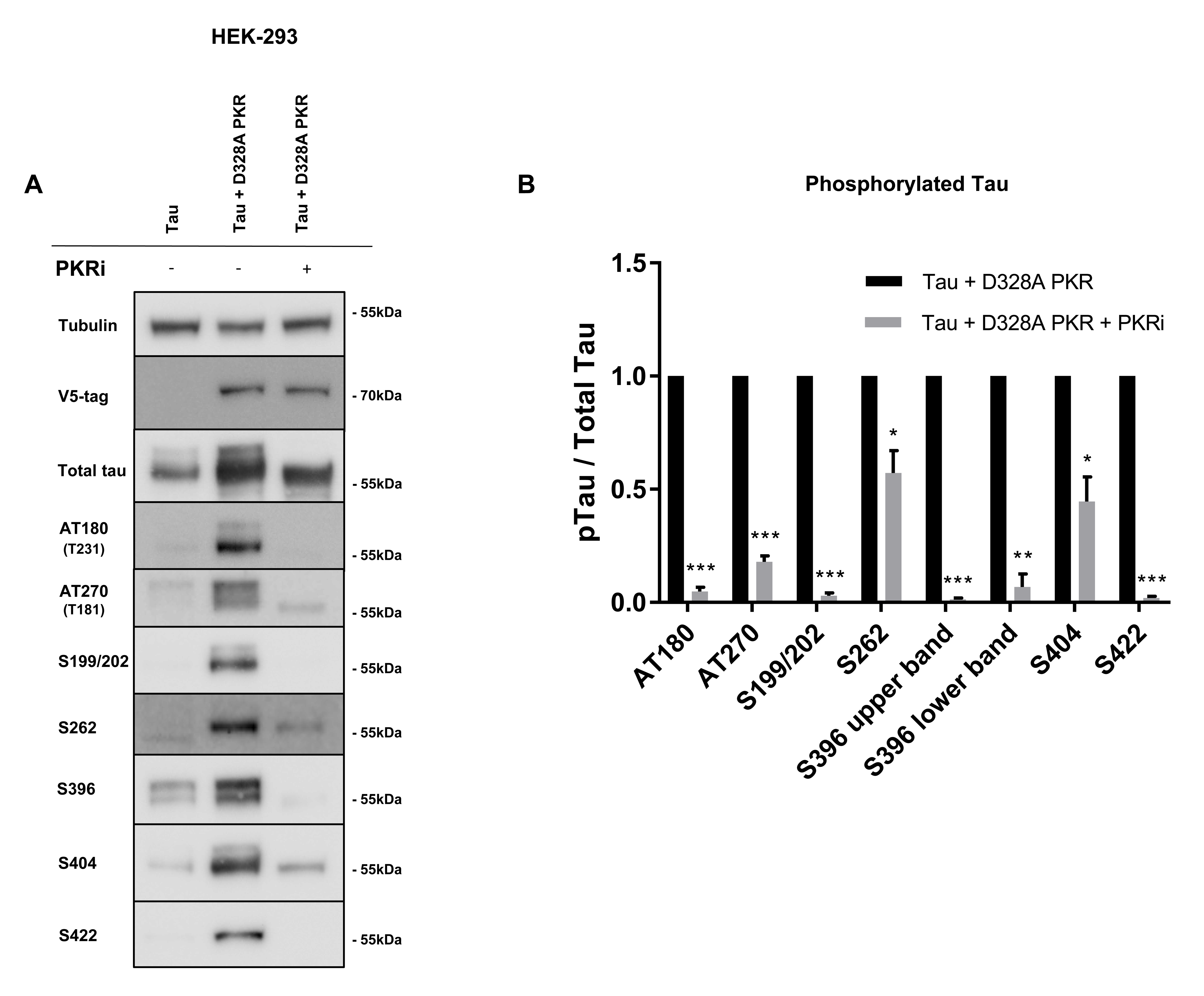

Supplement: Supplementary file 3 — Figure S3. PKR inhibition reverts tau phosphorylations induced by D328A PKR. A. Immunoblot of whole‐cell lysates from HEK 293T cells using total‐ and pTau‐specific antibodies as well as anti‐V5 tag to detect V5‐tagged constitutively active D328A PKR. Cells were co‐transfected with vectors expressing 2N4R tau and TD Tomato or 2N4R tau and V5‐tagged constitutively active D328A PKR for 24 h. Cells were treated with PKRi or DMSO as vehicle control for the last 4 h prior to cell lysis. Figure is representative of three independent experiments. B. Quantifications of the effect of PKR inhibition on different phospho‐tau epitopes/total tau in cells co‐expressing 2N4R tau and V5‐tagged constitutively active D328A PKR. Results from three independent experiments (n = 3, *P < 0.05, student t‐test). [file BPA-31-103-s005.tif]

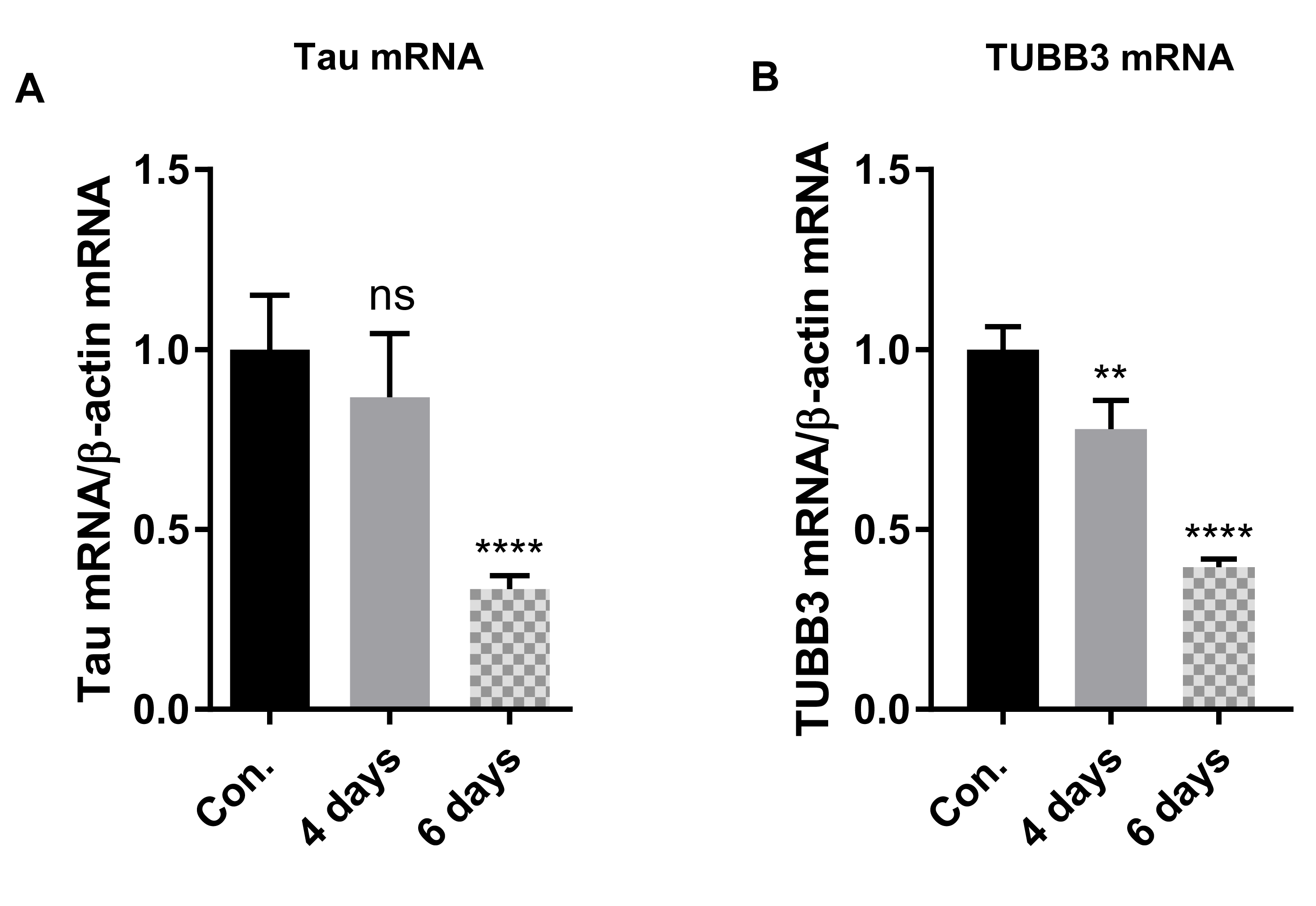

Supplement: Supplementary file 4 — Figure S4. Brain infection with Langat virus in C57BL/6J mice decreases mRNA from neuronal expressed proteins. (A,B) mRNA levels, in RNA extracted from brain homogenates, were quantified by qPCR. Tau mRNA (A) and neuron‐specific class III beta‐tubulin (TUBB3) mRNA (B) were normalized to β‐actin mRNA. The values in the figure exhibit the ratio between PBS injected mice and four‐ or 6 days Langat virus injected mice (n (PBS) = 4, n (4 days Langat virus) = 4 and n (6 days Langat virus) = 4, **P < 0.01, ****<0.0001 student t‐test). [file BPA-31-103-s001.tif]

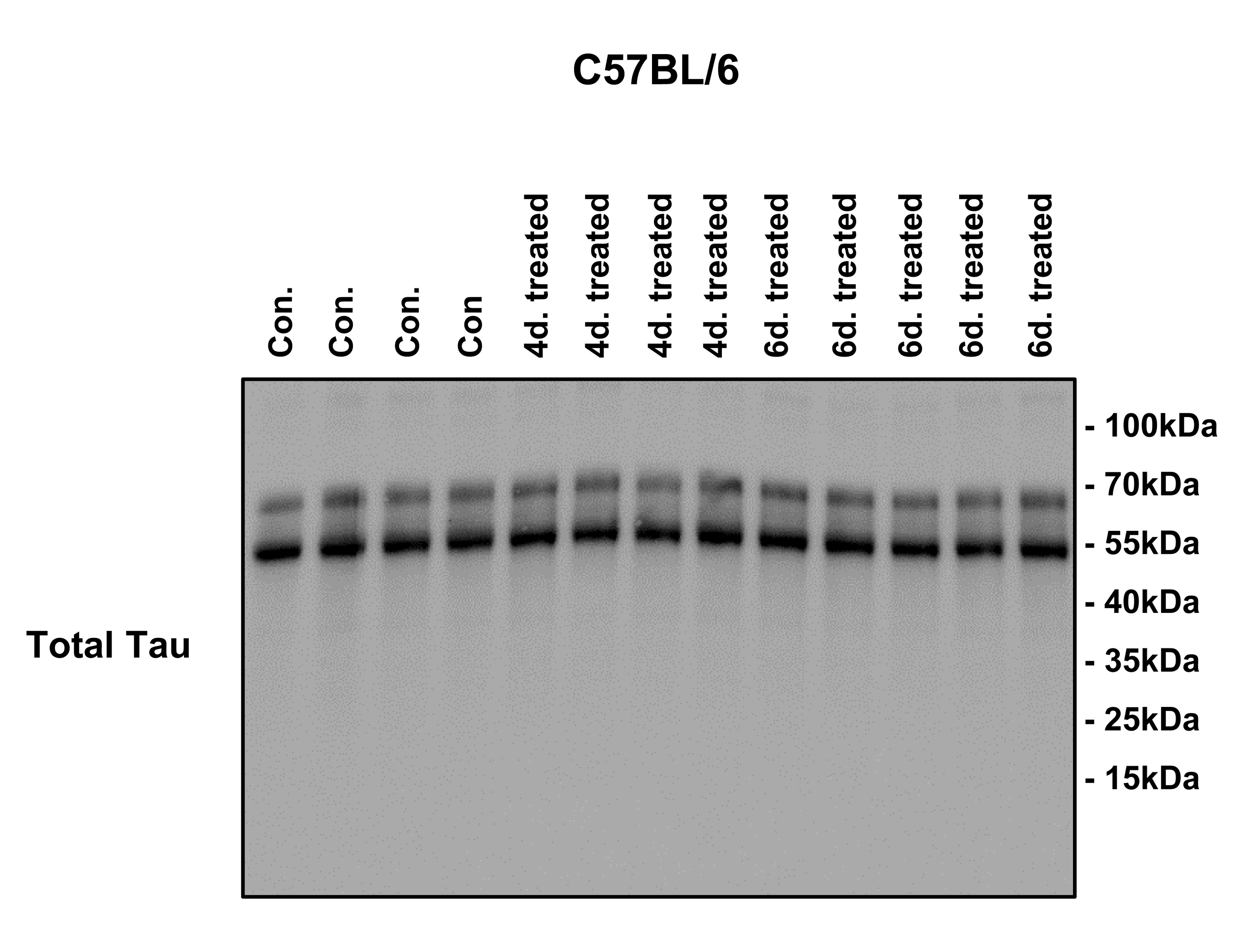

Supplement: Supplementary file 5 — Figure S5. Total tau antibody does not detect truncated species. Immunoblot of total brain homogenate from C57BL/6J mice intracranial injected with PBS or 100 FFU of Langat virus for 4‐ or 6 days. using in house‐produced total tau antibody. [file BPA-31-103-s002.tif]

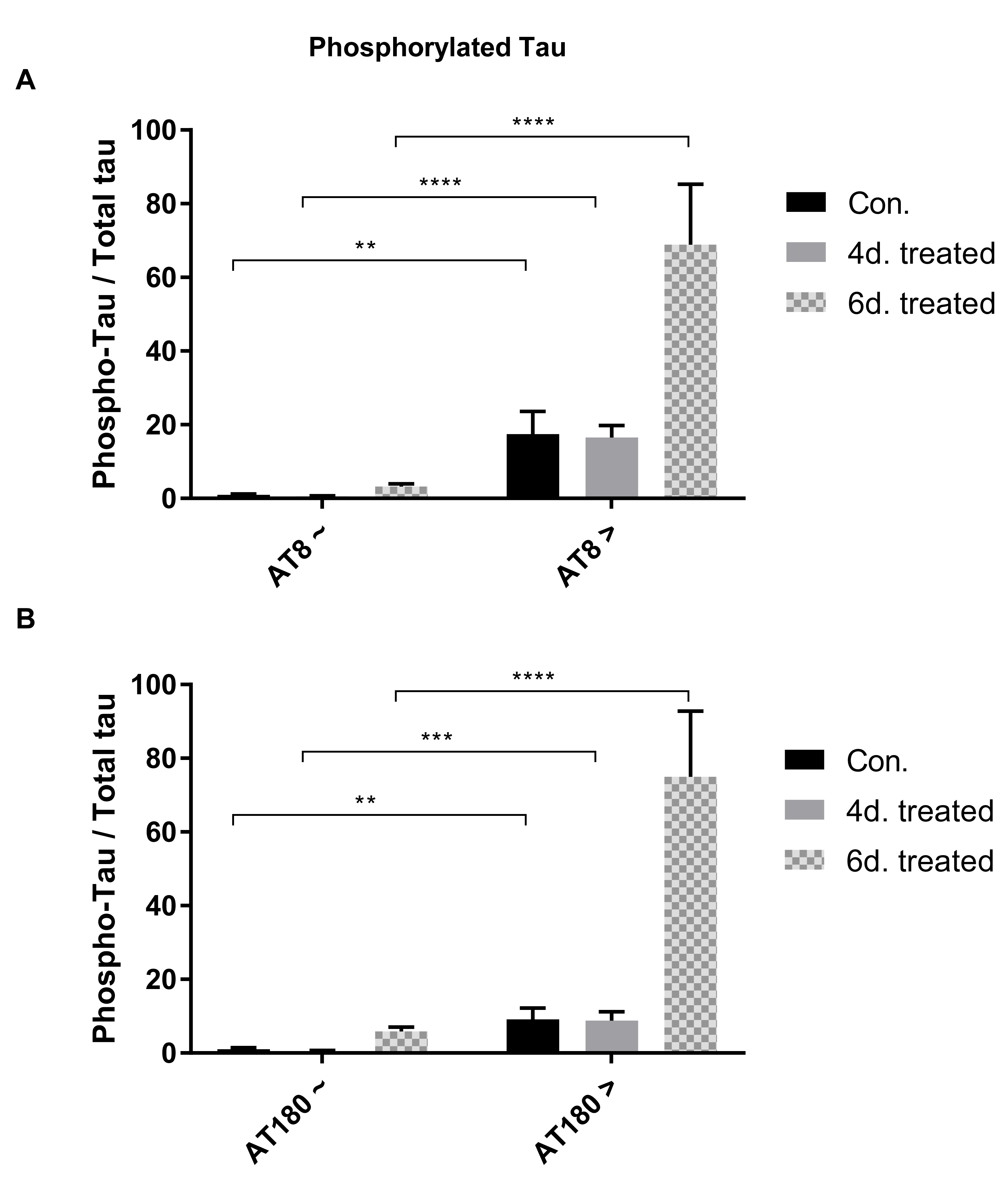

Supplement: Supplementary file 6 — Figure S6. Truncated tau is more extensively phosphorylated relative to full‐length tau. Quantifications of full‐length (~) and truncated (>) phospho‐tau epitopes (A) AT8 and (B) AT180 relative to their respective total tau signal and normalized to full‐length control. Difference in the fraction of phosphorylated full‐length and truncated tau was quantified for PBS‐treated controls, 4 d‐treated and 6 d‐treated mice (n (PBS) = 4, n (4 days Langat virus) = 4 and n (6 days Langat virus) = 5, **P < 0.01, ***<0.001, ****<0.0001 student t‐test). [file BPA-31-103-s003.tif]

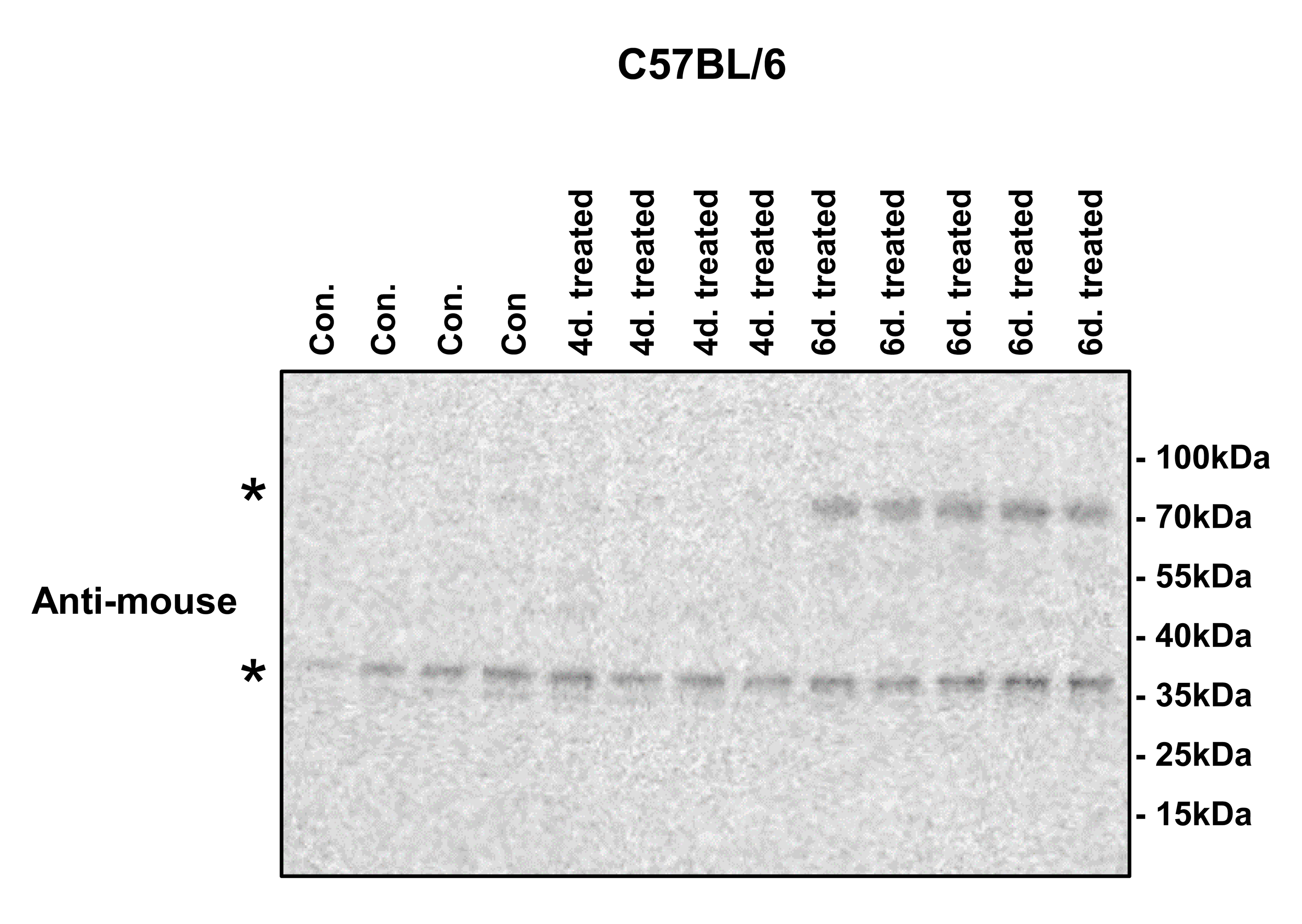

Supplement: Supplementary file 7 — Figure S7. Incubation with secondary mouse antibody alone detects two non‐tau related bands in mice brain homogenates. Immunoblot of total brain homogenate from C57BL/6J mice using secondary mouse antibody alone. Mice were intracranial injected with PBS or 100 FFU of Langat virus for 4‐ or 6 days. Two species of roughly 35 and 70 kDa in size, unrelated to phospho‐tau, were detected. * indicates size of non‐tau bands recognized by anti‐mouse secondary antibody. [file BPA-31-103-s004.tif]
